# Multiscale Metabolic Modeling of C4 Plants: Connecting Nonlinear Genome-Scale Models to Leaf-Scale Metabolism in Developing Maize Leaves

**DOI:** 10.1371/journal.pone.0151722

**Published:** 2016-03-18

**Authors:** Eli Bogart, Christopher R. Myers

**Affiliations:** 1 Laboratory of Atomic and Solid State Physics, Cornell University, Ithaca, NY, United States of America; 2 Institute of Biotechnology, Cornell University, Ithaca, NY, United States of America; Universidade Federal de Vicosa, BRAZIL

## Abstract

C4 plants, such as maize, concentrate carbon dioxide in a specialized compartment surrounding the veins of their leaves to improve the efficiency of carbon dioxide assimilation. Nonlinear relationships between carbon dioxide and oxygen levels and reaction rates are key to their physiology but cannot be handled with standard techniques of constraint-based metabolic modeling. We demonstrate that incorporating these relationships as constraints on reaction rates and solving the resulting nonlinear optimization problem yields realistic predictions of the response of C4 systems to environmental and biochemical perturbations. Using a new genome-scale reconstruction of maize metabolism, we build an 18000-reaction, nonlinearly constrained model describing mesophyll and bundle sheath cells in 15 segments of the developing maize leaf, interacting via metabolite exchange, and use RNA-seq and enzyme activity measurements to predict spatial variation in metabolic state by a novel method that optimizes correlation between fluxes and expression data. Though such correlations are known to be weak in general, we suggest that developmental gradients may be particularly suited to the inference of metabolic fluxes from expression data, and we demonstrate that our method predicts fluxes that achieve high correlation with the data, successfully capture the experimentally observed base-to-tip transition between carbon-importing tissue and carbon-exporting tissue, and include a nonzero growth rate, in contrast to prior results from similar methods in other systems.

## Introduction

C4 photosynthesis is an anatomical and biochemical system which improves the efficiency of carbon dioxide assimilation in plant leaves by restricting the carbon-fixing enzyme Rubisco to specialized bundle sheath compartments surrounding the veins, where a high-CO_2_ environment is maintained that favors CO_2_ over O_2_ in their competition for Rubisco active sites, thus suppressing photorespiration [1]. C4 plants are geographically and phylogenetically diverse, and represent the descendants of over 60 independent evolutionary origins of the system [2]. They include major crop plants such as maize, sugarcane and sorghum as well as many weeds and, relative to non-C4 (C3) plants, typically show improved nitrogen and water use efficiencies [3]. The agricultural and ecological significance of the C4 system and its remarkable convergent evolution have made it the object of intense study. The core biochemical pathways are now generally understood [4] but many areas of active research remain, including the genetic regulation of the C4 system [5], the importance of particular components of the system to its function (e.g., [6]), the significance of inter-specific variations in C4 biochemistry including alternative pathways for decarboxylation in the bundle sheath [7], details of the process of C4 evolution, [8–12] and the prospect of increasing yields of C3 crops by artificially introducing C4 functionality to those species [13, 14].

Mathematical modeling is a proven approach to gaining insight into C4 photosynthesis and will play an important role in addressing these questions. High-level, nonlinear models of photosynthetic physiology [15] relating enzyme activities, light and atmospheric CO_2_ levels, and the rates of CO_2_ assimilation by leaves have been widely applied to infer biochemical properties from macroscopic experiments and explore the responses of C4 plants under varying conditions. (We describe these models as ‘high-level’ since they describe in detail only a few of the individual biochemical reactions involved in the physiological processes they model, thus operating at a higher level of abstraction than more detailed kinetic models or genome-scale metabolic reconstructions.) More recently, detailed kinetic models have been used to explore the optimal allocation of resources to enzymes in an NADP-ME type C4 plant [16] and the relationship between the three decarboxylation types [17].

Large-scale constraint-based metabolic models offer particular advantages for the investigation of connections between the C4 system and a plant’s metabolism more broadly (for example, partitioning of nonphotosynthetic functions between mesophyll and bundle sheath, or the evolutionary recruitment of nonphotosynthetic reactions into the C4 cycle) and for interpreting high-throughput experimental data from C4 systems. The standard approach for predicting reaction rates in such models, flux balance analysis (FBA), determines predicted reaction rates *v*_1_, *v*_2_, … *v*_*N*_ by optimizing a biologically relevant function of the rates subject to the requirement that the system reach an internal steady state,

$$\begin{array}{c} \begin{array}{cccc} & \max_{\left(v_{1},v_{2},\ldots ,v_{N}\right)\in R^{N}} & & f(v)\\ & \text{s.t.} & & S\cdot v=0, \end{array} \end{array}$$ (1)

where the stoichiometry matrix *S* is determined by the network structure [18]. However, it is difficult to incorporate in these calculations the relationship between the rate *v*_*c*_ of carbon fixation by Rubisco and the rate *v*_*o*_ of the Rubisco oxygenase reaction, which depends nonlinearly on the ratio of the local oxygen and carbon dioxide concentrations (here expressed as equivalent partial pressures),

$$\begin{array}{c} \frac{v_{o}}{v_{c}}=\frac{1}{S_{R}}\frac{P_{O2}}{P_{CO2}} \end{array}$$ (2)

where *S*_*R*_ is the specificity of Rubisco for CO_2_ over O_2_. In the C4 case, the CO_2_ level in the bundle sheath compartment is itself a function of the rates of the reactions of the C4 carbon concentration system and the rate of diffusion of CO_2_ back to the mesophyll.

With the addition of Eq (2), the problem Eq (1) becomes nonlinear and cannot be solved with typical FBA tools, which use linear programming methods to analyze a feasible flux cone that is a convex polytope. Instead, the resulting problem is nonconvex [19] and a general-purpose nonlinear programming algorithm is required to numerically solve it. Such methods are more time-consuming and require additional care to ensure convergence to an appropriate solution.

While a number of prior constraint-based models of plant metabolism have described photosynthesis in some detail (e.g, [20–22], among others), such models have typically either ignored the constraint Eq (2) or assumed the oxygen and carbon dioxide levels *P*_*O*2_ and *P*_*CO*2_ are known and fixed *v*_*o*_/*v*_*c*_ accordingly. This approach is suitable for mature C4 leaves under many conditions (as well as photosynthetic microorganisms with carbon-concentrating mechanisms), where *v*_*o*_/*v*_*c*_ is approximately zero, or mature C3 leaves, where *v*_*o*_/*v*_*c*_ can often be readily predicted from environmental conditions. It cannot be applied, however, to some of the most interesting targets for simulation: developing tissue, mutants, and C3-C4 intermediate species, where *P*_*CO*2_ in the bundle sheath compartment is not necessarily high.

In other recent work, a high-level physiological model was used to determine *v*_*o*_, *v*_*c*_, and other key reaction rates given a few parameters, which were then fixed in order to solve Eq (1) [11]. This method yields realistic solutions, but its application is limited by the lack of a way to set the necessary phenomenological parameters (e.g., the maximum rate of PEP regeneration in the C4 cycle) based on lower-level, per-gene data (e.g., from transcriptomics or experiments on single-gene mutants).

Here, we treat the problem in a more general way by incorporating the nonlinear constraint Eq (2) directly into the optimization problem Eq (1) and solving the resulting nonlinear program numerically with the IPOPT package [23], using a new computational interface that we have developed, which allows rapid, interactive development of nonlinearly-constrained FBA problems from metabolic models specified in SBML format [24]. These computational tools in principle allow the incorporation of appropriate nonlinear kinetics into any existing FBA model.

We demonstrate the approach using a new genome-scale reconstruction of the metabolic network of *Zea mays*, developed with particular attention to photosynthesis and related processes, and confirm that the technique reproduces the nonlinear responses of well-validated, high-level physiological models of C4 photosynthesis [15], while also providing detailed predictions of fluxes throughout the network.

As noted above, FBA relies on the specification of a relevant objective function that is to be optimized through the appropriate distribution of metabolic fluxes. In the application of FBA to single-celled organisms, the traditional objective function chosen has been the rate of biomass production, under the assumption that an organism that is able to grow (and divide) most quickly will have a fitness advantage over others in a population. As constraint-based models and FBA have been extended to the realm of multicellular organisms, or to particular subsystems (pathways, tissues, organs, etc.), a challenge for the metabolic modeling field broadly has been to identify appropriate objective functions for use in FBA. In this work, we are using a metabolic model to explore the metabolism of a developing leaf. What is an appropriate objective function for this complex biological subsystem? The photosynthetically mature part of a leaf is presumably organized to some degree to assimilate CO_2_ at a high rate, but the metabolism of the developing, immature base is more devoted to cellular growth and differentiation. Our perspective is that different choices of objective functions enable us to probe different aspects of leaf physiology, by asking what metabolic flux distributions are most consistent with CO_2_ assimilation, biomass production, or agreement with experimental data.

**Fig 1:**
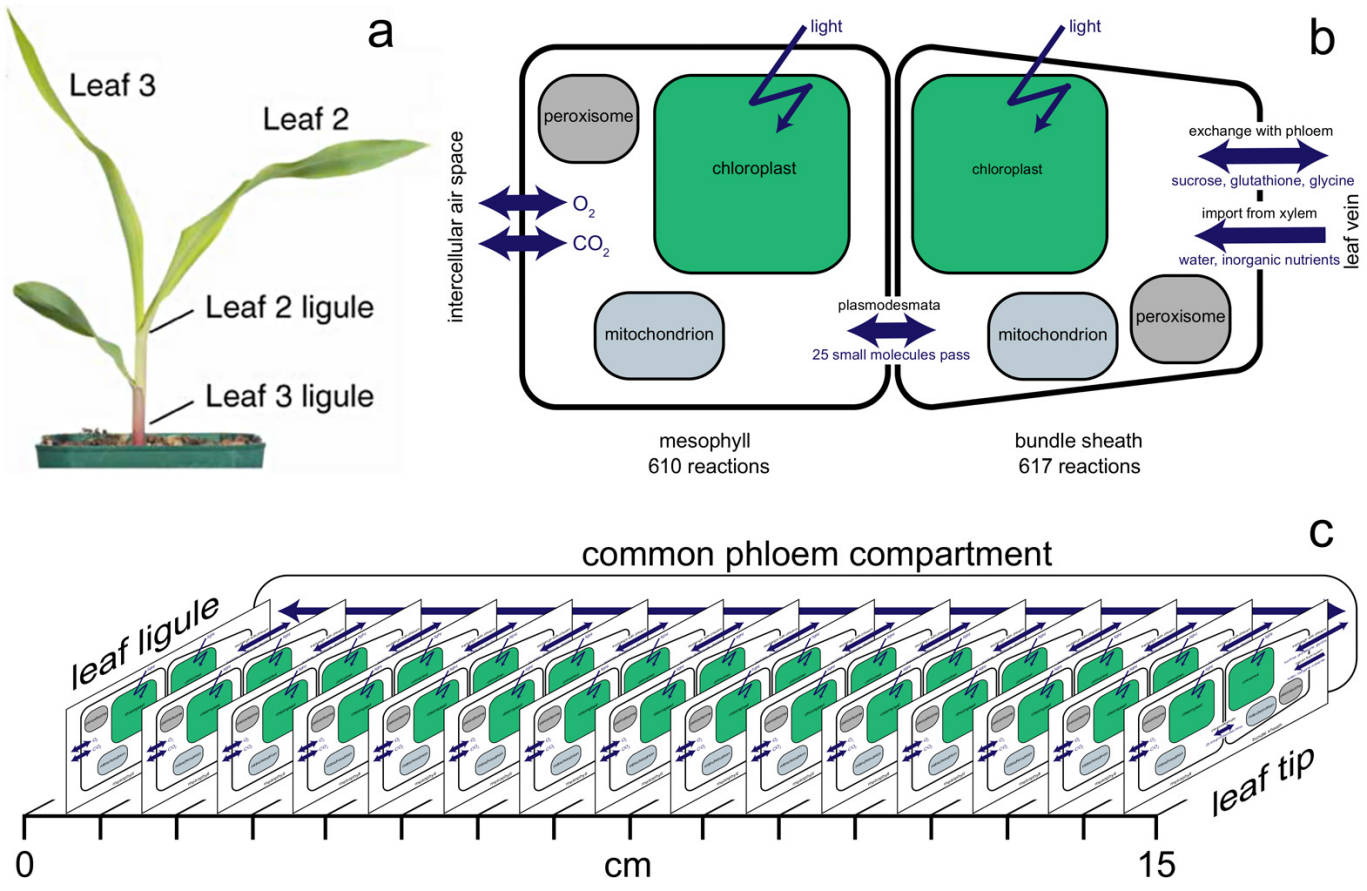
Maize plant and models. (a) Nine-day-old maize plant (image from [25]). (b) Organization of the two-cell-type metabolic model, showing compartmentalization and exchanges across mesophyll and bundle sheath cell boundaries. (c) Combined 121-compartment model for leaf 3 at the developmental stage shown in (a). Fifteen identical copies of the model shown in (b) represent 1-cm segments from base to tip.

With that preface, in this paper we attempt to use the combined results of enzyme assay measurements and multiple RNA-seq experiments to to infer the metabolic state at points along a developing maize leaf (Fig 1a). Although methods of flux prediction based on gene expression data have generally performed poorly, we hypothesize that expression and flux may be more tightly coupled in this system, which motivates the development of a new method, based on an objective function that rewards consistency between the pattern of expression change along the developing leaf and the pattern of flux change along the leaf for each reaction. With this approach, we predict reaction rates in a model of mesophyll and bundle sheath tissue in fifteen segments of the leaf, interacting through vascular transport of sucrose, glycine, and glutathione. We compare our predictions to results from radiolabeling experiments.

## Results

### Metabolic reconstruction of *Zea mays*

A novel genome-scale metabolic model was generated from version 4.0 of the CornCyc metabolic pathway database [26] and is presented in two forms. The comprehensive reconstruction involves 2720 reactions among 2725 chemical species, and incorporates CornCyc predictions for the function of 5204 maize genes, with 2064 reactions associated with at least one gene. A high-confidence subset of the model, excluding many reactions not associated with manually curated pathways or lacking computationally predicted gene assignments as well as all reactions which could not achieve nonzero flux in FBA calculations, involves 635 reactions among 603 species, with 469 reactions associated with a total of 2140 genes.

Both the comprehensive and high-confidence models can simulate the production of all major maize biomass constituents (including amino acids, nucleic acids, fatty acids and lipids, cellulose and hemicellulose, starch, other carbohydrates, and lignins, as well as chlorophyll) under either heterotrophic or photoautotrophic conditions and include chloroplast, mitochondrion, and peroxisome compartments, with key reactions of photosynthesis (including a detailed representation of the light reactions), photorespiration, the NADP-ME C4 cycle, and mitochondrial respiration localized appropriately. Gene associations for reactions present in more than one subcellular compartment have been refined based on the results of subcellular proteomics experiments and computational predictions (as collected by the Plant Proteomics Database [27]) to assign genes to reactions in appropriate compartments.

Two alternative sets of biomass production reactions are incorporated in the model. One system (based closely on iRS1563 [22]) allows the production of biomass components only in a fixed ratio (as is appropriate in FBA calculations that maximize biomass production.) The other set of reactions allows individual biomass components to be produced without any constraint on their rates, and is used in some calculations below to allow shifts in biomass composition along the leaf developmental gradient to be predicted based on experimental data.

A model for interacting mesophyll and bundle sheath tissue in the leaf was created by combining two copies of the high-confidence model, with transport reactions to represent oxygen and CO_2_ diffusion and metabolite transport through the plasmodesmata, and restricting exchange reactions appropriately (nutrient uptake from the vascular system to the bundle sheath, and gas exchange with the intercellular airspace to the mesophyll). A schematic of the two-cell model is shown in Fig 1b.

Both single-cell versions of the model and the two-cell model, designated iEB5204, iEB2140, and iEB2140x2 respectively (based on the primary author’s initials and number of genes included, according to the established naming convention [28]), are available in SBML format (S1–S3 Models.)

### Nonlinear flux-balance analysis

To solve nonlinear optimization problems incorporating the constraints discussed above, we developed a Python package which—given a model in SBML format, arbitrary nonlinear constraints, a (potentially nonlinear) objective function, and all needed parameter values—infers the conventional FBA constraints of Eq (1) from the structure of the network, automatically generates Python code to evaluate the objective function, all constraint functions, and their first and second derivatives, and calls IPOPT through the pyipopt interface [29]. Source code for the package is available in S1 Protocol and online (http://github.com/ebogart/fluxtools).

**Fig 2:**
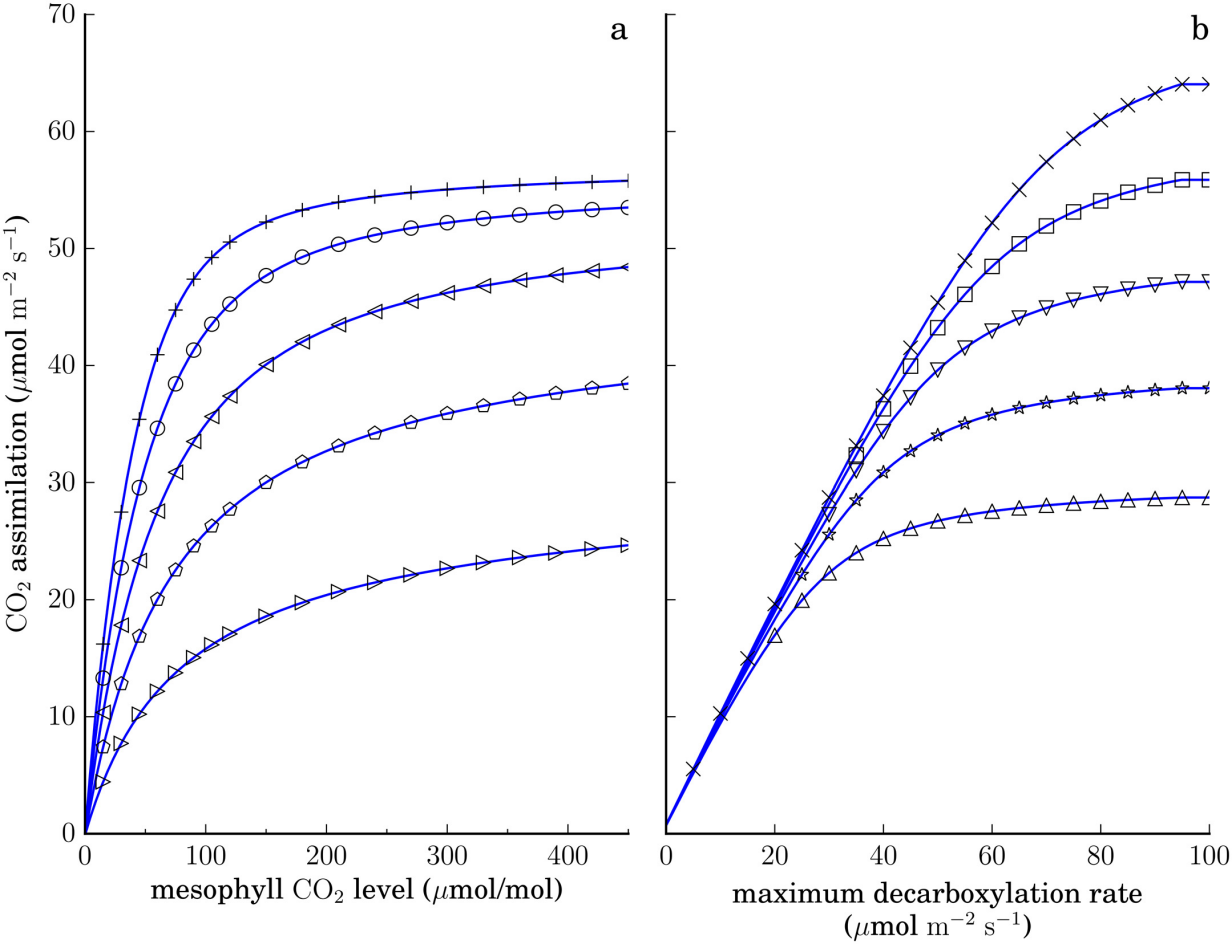
CO_2_ assimilation rates (*A*) predicted by the C4 photosynthesis model of [15], solid lines, and the present nonlinear genome-scale model (markers) maximizing CO_2_ assimilation with equivalent parameters. The nonlinear model incorporates the mesophyll CO_2_ level as a parameter through the constraints in Eqs 5, 6 and 7. Left, *A* vs mesophyll CO_2_ levels with varying PEPC levels (top to bottom, *v*_*p*,max_ = 110, 90, 70, 50, and 30 μmol m^-2^ s^-1^). Right, *A* vs total maximum activity of all bundle sheath decarboxylase enzymes (equivalent to the maximum PEP regeneration rate *V*_*pr*_ in [15]) at varying Rubisco levels (top to bottom, *v*_*c*,max_ = 70, 60, 50, 40, and 30 μmol m^-2^ s^-1^). Other parameters as in Table 4.1 of [15], except with nonphotorespiratory respiration rates *r*_*d*_ = *r*_*m*_ = 0.

As a validation of this nonlinear optimization approach (as well as the two-cell-type model described above), Fig 2 demonstrates that, if we choose an objective function so as to maximize the rate of CO_2_ assimilation with nonlinear kinetic constraints [Eqs (5), (6), (7) below] our model produces predictions consistent with the results of the physiological model of [15]. Note that the effective value of one macroscopic physiological parameter may be governed by many microscopic parameters in the genome-scale model. In the figure, the effective maximum PEP regeneration rate *V*_*pr*_ is controlled by the maximum rate of three decarboxylase reactions in the bundle sheath compartment, but with an appropriate choice of parameter values any of at least 10 reactions of the C4 system could become the rate-limiting step in PEP regeneration, and in the calculations below, expression levels for any of the 42 genes associated with these reactions (S1 Table) could influence the net PEP regeneration rate. (A fixed biomass composition is used in these calculations; sucrose is also allowed to be exported freely, so assimilated carbon may be directed to either sucrose or biomass production.)

### Flux predictions in the developing leaf based on multiple data channels

Maize leaves display a developmental gradient along the base-to-tip direction, with young cells in the immature base and fully differentiated cells at the tip [25, 30]. This developmental gradient has recently been studied experimentally with great spatial resolution, identifying changes in gene expression from leaf base to tip and in cell-type specificity of expression. We are particularly interested in quantitative changes in metabolic enzyme expression along this gradient, and the impact of those changes on the leaf metabolic state. We have therefore combined the RNA-seq datasets of Wang et al. [31] and Tausta et al. [32] to estimate expression levels (as FPKM) for 39634 genes in the mesophyll and bundle sheath cells at 15 locations along the developmental gradient, representing 1 cm segments of the third leaf of a 9-day-old maize plant. The combined dataset provides expression information for 920 reactions in the two-cell model (460 each in mesophyll and bundle sheath cells).

A whole-leaf metabolic model, iEB2140x2x15, was created from fifteen copies of the two-cell model, each representing a 1-cm segment, interacting through the exchange of sucrose, glycine, and glutathione through a common compartment representing the phloem. The resulting 121-compartment model, Fig 1c, involves 18780 reactions among 16575 metabolites.

As noted above, the large-scale transcriptional reprogramming that takes place along the developing leaf makes specification of a single, biologically relevant objective function not obvious. Therefore, we have constructed an objective function aimed at identifying flux distributions that are most consistent with the transcriptional variation occurring along the leaf. Subject to the requirements that reaction rates in each of the 15 segments obey both the FBA steady-state constraints (Eq 1) and the constraints governing Rubisco and PEPC kinetics and CO_2_ diffusion (Eqs 5, 6 and 7, presented in detail below) we determined the set of rates *v*_*ij*_ for each reaction *i* at each segment *j* which were most consistent with the base-to-tip variation in the gene expression data, by minimizing the objective function

$$\begin{array}{c} F\left(v\right)=\sum_{i=1}^{N_{r}}\sum_{j=1}^{15}\frac{{\left(e^{s_{i}}\left|v_{ij}\right|-d_{ij}\right)}^{2}}{\delta_{ij}^{2}}+\alpha \sum_{i=1}^{N_{r}}s_{i}^{2} \end{array}$$ (3)

where *N*_*r*_ = 920 is the number of reactions associated with at least one gene present in the expression data, *d*_*ij*_ and *δ*_*ij*_ are the expression data and associated experimental uncertainty for reaction *i* at leaf segment *j*, and *s*_*i*_ is an optimizable scale factor associated with reaction *i*. This objective function was used in all the calculations presented below, except where specifically noted.

Effectively, this method—similar to the method of Lee et al. [33] or FALCON [34]—performs a constrained least-squares fit of the fluxes to the expression data. While the flux through a reaction catalyzed by an enzyme need not correlate with the expression level of the genes encoding the enzyme, we hypothesized that this approach could be well-suited to the leaf developmental gradient in particular, as discussed in detail below.

Allowing the scale factors *s*_*i*_ to vary emphasizes agreement between fluxes and data in their trend along the developmental gradient, rather than in their absolute value: if the data associated with reaction *R*_*i*_ has average value 100 FPKM, a solution in which *R*_*i*_ has mean flux 10 μmol m^-2^ s^-1^ but correlates well with the data can achieve (with appropriate choice of scale factor) a lower cost than a solution in which *R*_*i*_ has mean flux 100 μmol m^-2^ s^-1^ but is anticorrelated. The penalty term $\alpha \sum s_{i}^{2}$ favors solutions in which, generally, reactions with larger associated expression data carry higher fluxes. In the current work, these criteria were weighted equally, with the tradeoff parameter *α* set to 1. We require *s*_*a*_ = *s*_*b*_ if reactions *a* and *b* are mesophyll and bundle sheath instances of the same reaction.

To constrain the overall scale of the fluxes and further improve accuracy, we incorporated available enzyme activity assay data from [31] for seventeen enzymes (including Rubisco and PEPC) along the 15 leaf segments as additional constraints on the optimization problem, requiring for each enzyme *k* and segment *j*

$$\begin{array}{c} E_{jk}\geq \left|v_{k1}\right|+\ldots +\left|v_{kn}\right| \end{array}$$ (4)

where *E*_*jk*_ is the measured maximal activity of the enzyme at that segment and the sum on the right hand side includes all the reactions which represent enzyme *k* in the mesophyll, bundle sheath, and subcompartments of those cells if applicable.

Solving the optimization problem yielded predictions for reaction rates and other variables (S2 Table). Upper and lower bounds on selected variables (S3 Table) were determined through a modified flux variability analysis (FVA) procedure [35] described in S2 Appendix.

#### Predicted source-sink transition

**Fig 3:**
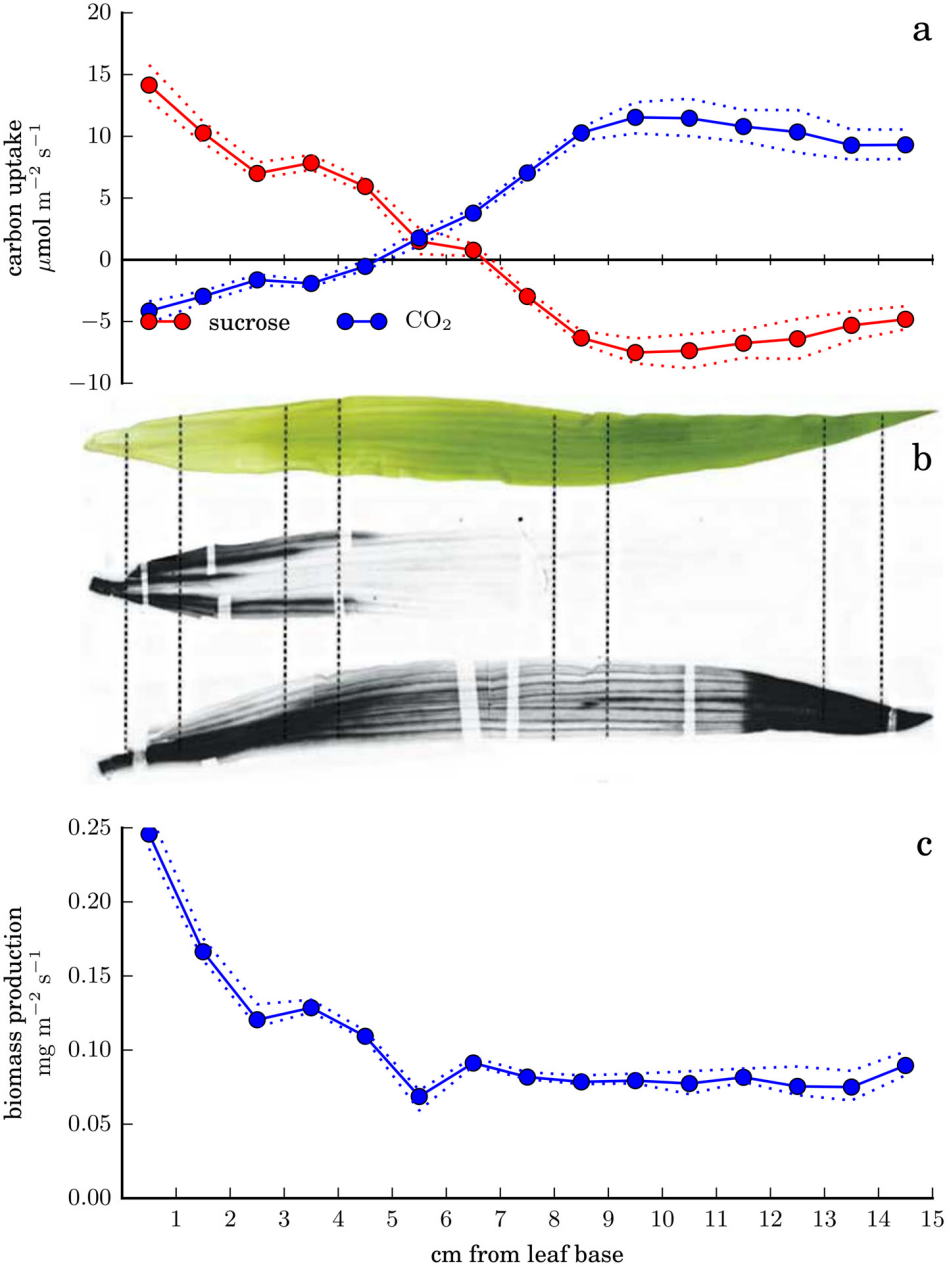
Source-sink transition along the leaf as predicted by optimizing the agreement between fluxes in the nonlinear model and RNA-seq data. Predicted fluxes are obtained by minimizing the objective function of Eq 3. (a) Predicted rates of exchange of carbon with the atmosphere and phloem along the leaf. (b) Experimental observation of the source-sink transition, reproduced from [25]. Upper image, photograph of leaf 3; middle image, autoradiograph of leaf 3 after feeding ^14^CO_2_ to leaf 2; lower image, autoradiograph of leaf 3 after feeding ^14^CO_2_ to the tip of leaf 3. (c) Total biomass production in the best-fitting solution. In panels a and c, dotted lines indicate minimum and maximum predicted rates consistent with an objective function value no more than 0.1% greater than the optimal value. Here, the biomass composition is allowed to vary along the leaf; S8 Fig shows corresponding results where the biomass composition is fixed.

As shown in Fig 3, in the outer, more photosynthetically developed, portion of the leaf, our optimal fit predicts net CO_2_ uptake, with most of the assimilated carbon incorporated into sucrose and exported to the phloem. Near the base of the leaf, sucrose is predicted to be imported from the phloem and used to drive a high rate of biomass production, with some concomitant net release of CO_2_ to the atmosphere by respiration.

This transition between a carbon-exporting source region and a carbon-importing sink region is well known, and the predicted transition point between the two, approximately 6 cm above the base of the leaf, can be compared to the ^14^C-labeling results of Li et al. [25] in the same experimental conditions. Fig 3b shows the location of labeled carbon in leaf 3 after feeding labeled CO_2_ to leaf 2 (center image) or leaf 3 (bottom image, with the dark region above 11.5 cm showing where label was supplied). Li et al. [25] identified the sink region as the lowest 4 cm of the leaf; the transition is not perfectly sharp and quantitative comparison of exchange fluxes is not possible, but the nonlinear FBA results appear to slightly overestimate the size of the sink region. (Note that these results do not allow direct assessment of spatial variation in the CO_2_ *uptake* rate.)

Agreement might be improved under a different assumption about net sucrose import or export by leaf 3 (here, we have assumed that the import visible in the center image is exactly balanced by the export suggested by the high density of labeled carbon at the absolute base in the lower image).

The net rate of CO_2_ assimilation predicted in the outer, most mature leaf segments, 8–11 μmol m^-2^ s^-1^, is lower than that typically measured in more mature maize plants (e.g., rates of 20–30 μmol m^-2^ s^-1^ in 22-day-old wild-type plants under comparable conditions [6]), but photosynthetic capacity may still be increasing even in these segments.

In addition to sucrose, glycine and glutathione are predicted to be exported from the source region through the phloem and reimported by the sink region, consistent with our expectations that nitrogen and sulfur reduction will occur preferentially in the photosynthesizing region (S1 Fig). Note that this behavior emerges from the data even though there is no explicit requirement in the model that net phloem transport occur in a basipetal direction.

#### Predicted C4 system function

**Fig 4:**
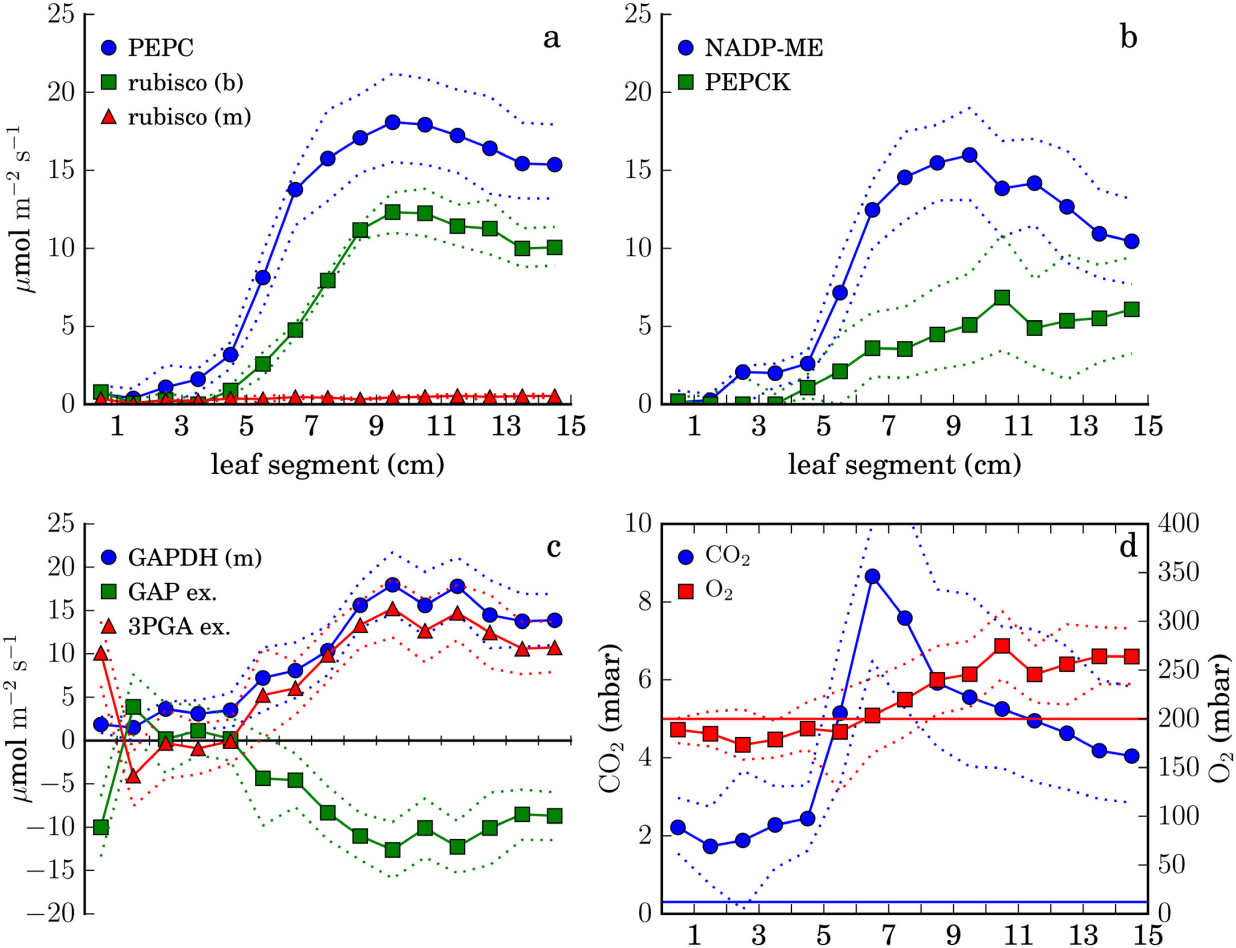
Operation of the C4 system in the best-fitting solution, as determined by minimizing the objective function, Eq 3. (a) Rates of carboxylation by PEPC in the mesophyll and Rubisco in the mesophyll and bundle sheath. (b) Rates of CO_2_ release by PEP carboxykinase and chloroplastic NADP-malic enzyme in the bundle sheath. (c) Transport of 3-phosphoglycerate and glyceraldehyde 3-phosphate from bundle sheath to mesophyll (or the reverse, where negative) and glyceraldehyde 3-phosphate dehydrogenation rate in the mesophyll chloroplast, showing the involvement of the mesophyll in the reductive steps of the Calvin cycle throughout the source region. (d) Oxygen and carbon dioxide levels in the bundle sheath. Straight lines show mesophyll levels. Throughout, dotted lines indicate minimum and maximum predicted values consistent with an objective function value no more than 0.1% greater than the optimal value.

Fig 4 shows predicted rates of key reactions of the C4 system and CO_2_ and O_2_ levels in the bundle sheath. As expected, the model predicts that a C4 cycle will operate in the source region of the leaf, elevating the CO_2_ level in the bundle sheath. The CO_2_ level is also elevated in the source region; this is an immediate consequence of respiration in the bundle sheath and Eq (7). It may be overestimated here because we have assumed a constant value for the bundle sheath CO_2_ conductance *g*_*s*_ (as measured by Bellasio et al. [36]); in fact, gene expression associated with synthesis of the diffusion-resistant suberin layer between bundle sheath and mesophyll peaks at 4 cm above the leaf base [31], *g*_*s*_ is presumably higher below that point.

In the Calvin cycle, most reactions are predicted to be bundle-sheath specific, but the reductive phase is active in both cells, with approximately half the 3-phosphoglycerate produced in the bundle sheath transported to the mesophyll and returned as dihydroxyacetone phosphate (Fig 4c); this is a known aspect of NADP-ME C4 metabolism connected to reduced photosystem II activity in the bundle sheath cells [37], which is also predicted here (S2 Fig). Consistent with conclusions drawn independently from the transcriptomic data, as well as proteomic data from the same system [25, 31, 38], the model does not predict a C3-like metabolic state as a developmental intermediate stage. As expected in maize [39], a significant role for phosphoenolpyruvate carboxykinase (PEPCK) as a decarboxylating enzyme operating in the bundle sheath in parallel with NADP-ME is predicted (Fig 4b).

While the predictions are generally consistent with the standard view of the C4 system in maize, there are minor discrepancies. In the mesophyll, our calculations predict that malate production occurs in the mitochondrion, rather than the chloroplast. In both mesophyll and bundle sheath, phosphoenolpyruvate is formed by pyruvate-orthophosphate dikinase (PPDK) in the chloroplast at a higher rate than necessary to sustain the C4 cycle; the excess is converted again to pyruvate by pyruvate kinase in the cytoplasm, with the resulting ATP consumed by the model’s generic ATPase reaction. Finally, in the bundle sheath, a modest rate of PEPC activity is predicted, recapturing CO_2_ only to have it released again by the decarboxylases (S3 Fig). Further refinement of the associations of genes to reactions in the model might resolve some of these discrepancies.

#### Global agreement between fluxes and data

**Fig 5:**
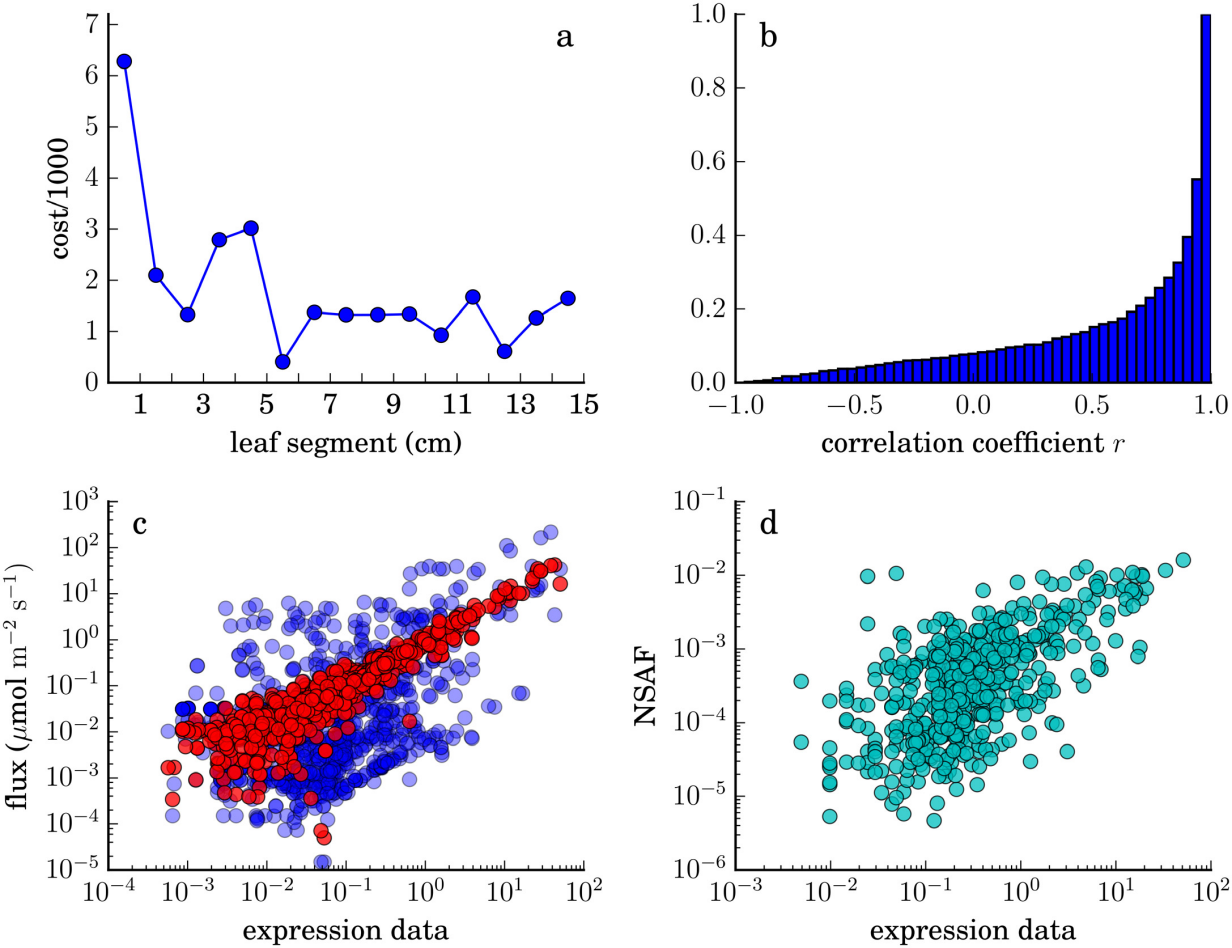
Agreement between RNA-seq data and predicted fluxes. (a) Contribution of each segment to the objective function (Eq (3), excluding costs associated with scale factors). (b) Cumulative histogram of Pearson correlations between data and predicted fluxes for all reactions. (c) Predicted fluxes versus expression data at the tip of the leaf (blue, raw fluxes; red, after rescaling each flux *v*_*i*_ by the optimal factor $e^{s_{i}}$ of Eq (3)). Some outliers with very low predicted flux are not shown. (d) Relationship between RNA-seq and proteomics measurements for 506 proteins in the 14th segment from the base, redrawn from the data of [40]. NSAF, normalized spectral abundance factor.

Fig 5 summarizes overall properties of the predicted fluxes. It is not clear why agreement between data and predicted fluxes is poorer at the base, as shown in Fig 5a. As discussed below, the cell-type-specific RNA-seq data from Tausta et al. [32] does not extend below the fourth segment from the base of the leaf; at the base we have assumed expression levels for all genes are equal in mesophyll and bundle sheath. Though proteomics experiments on the same system [38] generally found limited cell-type specificity at the leaf base, this assumption is likely an oversimplification, and could limit the ability of the algorithm to find a flux prediction consistent with the data there.

For most reactions, the correlation between the base-to-tip expression pattern and the base-to-tip trend in predicted flux is high. The cumulative histogram in Fig 5b shows that the Pearson correlation *r* > 0.92 for more than half of the reactions in the model with associated expression data.

Differences in expression levels between different reactions, however, correlate only weakly with the differences in fluxes between those reactions, as shown for segment 15 in Fig 5c (blue circles). After rescaling fluxes by the optimal per-reaction scale factors, a clear relationship emerges (Fig 5c, red circles), confirming that the scale factors are functioning as intended. Of course we should not expect a perfect correlation between data on transcript levels and predicted fluxes through associated reactions. The limited correlation between fluxes and expression data across different reactions presumably follows, in part, from the imperfect correlation between expression data and protein abundance across different genes, as illustrated in Fig 5d with data from the same experimental system [40], as well as from the different catalytic capabilities of different enzymes, posttranslational regulation, differences in substrate availability, etc.

#### Reconciling expression data and network structure

**Fig 6:**
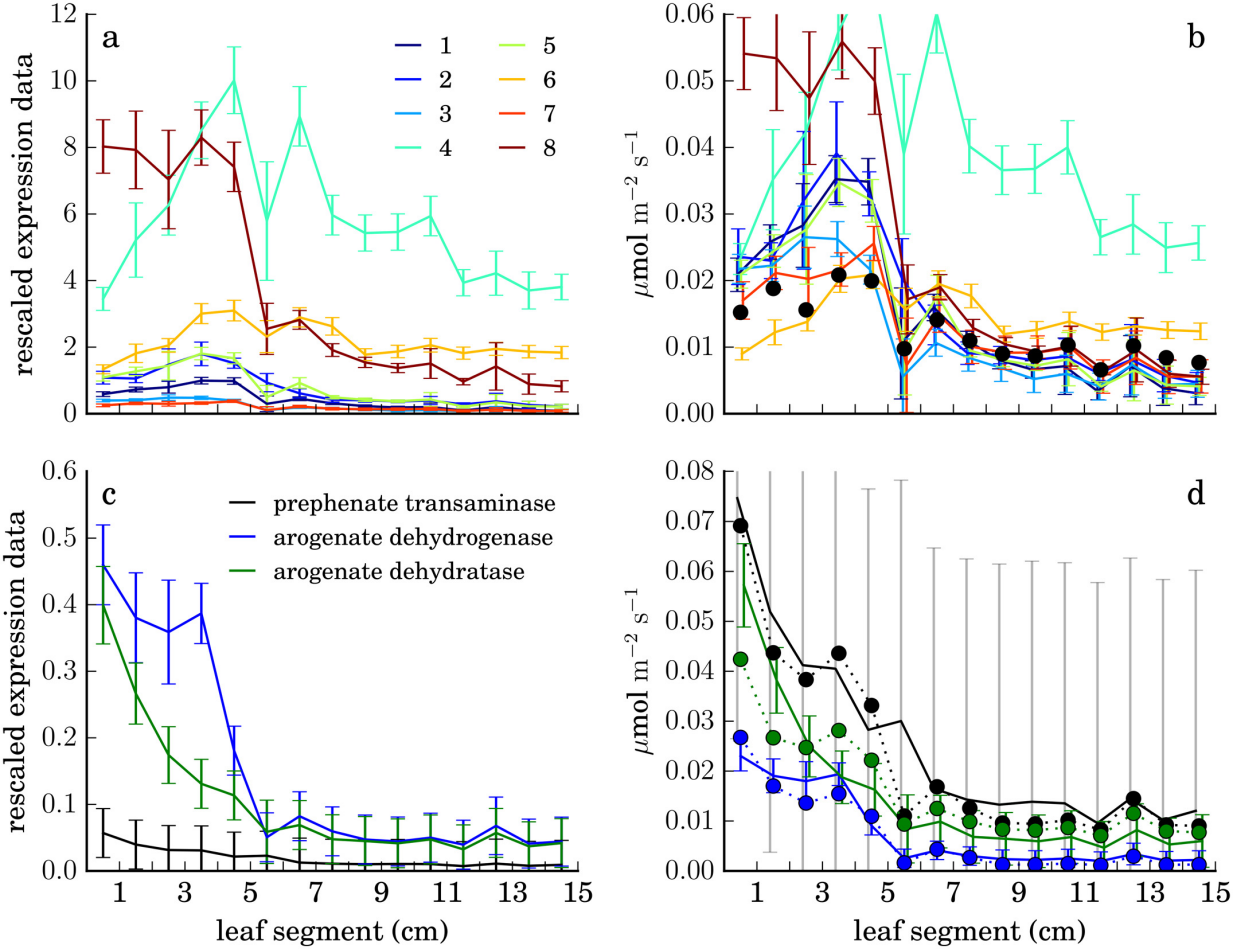
Comparison of RNA-seq data to predicted fluxes for a linear pathway and around a metabolic branch point. Upper panels, chlorophyllide a synthesis in the mesophyll; lower panels, production of arogenate in the bundle sheath by prephenate transaminase and its consumption by arogenate dehydrogenase and arogenate dehydratase. Left, aggregate RNA-seq data and experimental standard deviations for each reaction rescaled by a uniform factor (see text). Right, same data and errors further rescaled by reaction-specific optimal factors (*e*^−*si*^, in the variables of Eq 3) to best match data with predicted fluxes (solid circles). Fluxes are equal for all reactions of the linear pathway (1, uroporphyrinogen decarboxylase, 2, coproporphyrinogen oxidase, 3, protoporphyrinogen oxidase, 4, magnesium chelatase, 5, magnesium protoporphyrin IX methyltransferase, 6, magnesium protoporphyrin IX monomethyl ester cyclase, 7, divinyl chlorophyllide a 8-vinyl-reductase, 8, protochlorophyllide reductase.) Error bars represent standard deviations of expression measurements across multiple replicates.

Fig 6 illustrates the operation of the fitting algorithm in detail, using two regions of the metabolic network with simple structure as examples.

In Fig 6a, expression data for eight reactions of the pathway leading to chlorophyllide a are shown. Expression levels for the different reactions at any point on the leaf may span an order of magnitude or more, but the FBA steady-state assumption requires the rates of all reactions in this unbranched pathway to be equal at each point. (The branch leading to heme production is not included in the reconstruction.) Applying the optimal rescaling determined for each reaction’s expression data, shown in panel b, allows the flux prediction for the pathway (solid dots) to achieve reasonable agreement with the data. (Note that data for reaction 4 cannot be further scaled down because of the lower limit exp(−5) on its scale factor exp(*s*_4_), imposed for technical reasons.)

Fig 6c shows data for a three-reaction branch point in aromatic amino acid synthesis. To balance production and consumption of arogenate, the prephenate transaminase flux must equal the sum of the fluxes through arogenate dehydrogenase (to tyrosine) and arogenate dehydratase (to phenylalanine) but expression is consistently lower for the transaminase than the other enzymes. After rescaling (Fig 6d), the data agree well with the stoichiometrically consistent flux predictions (solid dots). The predicted ratio of dehydrogenase to dehydratase flux reflects data for downstream reactions.

#### Comparison to other methods for integrating RNA-seq data

S4 Fig shows predictions that result when the scale factors *s*_*i*_ of Eq (3) are fixed to zero. The source-sink transition is apparent but the C4 cycle operates at lower levels, the example pathways of Fig 6 (and a number of others) show little or no activity, and predicted fluxes along the leaf are not as tightly correlated with their associated expression data.

S5 Fig shows the metabolic state predicted by applying the expression data for each reaction as an upper bound on the absolute value of the reaction rate as in the E-Flux method [41] to the fifteen-segment model with the same RNA-seq data. (Here, the objective function maximizes CO_2_ assimilation.) The C4 system is predicted to operate, but no source-sink transition is apparent, and typical data-predicted flux correlations are poor. Imposing a realistic biomass composition restores the source-sink transition and somewhat improves correlation between data and fluxes (S6 Fig). Fluxes predicted by E-Flux are generally smaller than those predicted by the least-squares method, with or without per-reaction scale factors.

S9 Fig compares the fluxes predicted at the tip by optimizing agreement with the data through the non-biological objective function Eq (3), fluxes predicted at the tip with an explicit biological objective function (maximizing CO_2_ assimilation) constrained by the experimental data in the E-Flux method, and fluxes predicted in an FBA calculation which ignores the data entirely (minimizing total flux while achieving the same CO_2_ assimilation rate as predicted at the tip by the least-squares method.) Both data-integration methods lead to predictions very different from the unconstrained FBA calculation.

S10 Fig shows results obtained when the requirement that predicted fluxes obey the kinetic laws [Eqs (5), (6), (7)] is relaxed. The source-sink transition is still apparent and predictions for most reactions are similar, but quantitative and qualitative changes in predicted rates of several key reactions of the C4 system are observed.

## Discussion

### Fitting metabolic fluxes to expression data

The expression of a gene encoding a metabolic enzyme need not correlate with the rate of the reaction that enzyme catalyzes. The relationship between transcription and degradation of mRNA and control of flux is indirect, mediated by protein translation, folding, and degradation, complex formation, posttranslational modification, allosteric regulation, and substrate availability. Indeed, as reviewed by [42], experimentally observed correlations among RNA-seq or microarray data (each itself an imperfect proxy for mRNA abundance or transcription rate), protein abundance, enzyme activity, and fluxes are variable and often weak.

For example, RNA-seq and quantitative proteomic data obtained from maize leaves at the same developmental stage studied here, harvested simultaneously from plants grown together, showed Pearson correlation approximately 0.6 across the entire dataset, but some significantly lower values were found when correlations were restricted to genes of particular functional classes, and measured mRNA/protein ratios for individual genes varied up to 10-fold along the gradient [40]. A subset of this data is shown in Fig 5d.

The most comprehensive study of the issue in plants so far [43] found so little agreement between RNA-seq and 13C-MFA data from embryos of two *Brassica napus* accessions that the authors concluded the inference of central metabolic fluxes from transcriptomics is, in general, impossible.

In this light, it is not surprising that methods for integrating transcriptomic data with metabolic models to predict reaction rates have met with limited success. Machado and Herrgård [44] reviewed 18 such methods and assessed the performance of seven of them on three test datasets from *E. coli* and *Saccharomyces cerevisiae* where experimentally measured intracellular and extracellular fluxes were available for comparison. None of the methods consistently outperformed parsimonious FBA simulations which completely ignored transcriptomic data.

Nonetheless, we hypothesized that in the leaf developmental gradient system in particular, expression levels would correlate enough with fluxes to allow usable predictions to be made with a careful choice of objective function. Our justification for this hypothesis is twofold.

First, the metabolic transition between the heterotrophic sink region at the base and the photoautotrophic source region at the tip is particularly dramatic, involving a large number of reactions which are effectively absent in one region but carry high fluxes in the other [25]; so long as even a slight correlation between transcript levels and fluxes exists, such a reconfiguration should be apparent from expression data.

Second, although the developing maize leaf is biologically more complex than microbial growth experiments, the relationship between expression levels and fluxes may be actually be closer in the leaf. Leaf development is a stereotyped, frequently repeated, relatively slow, one-way process, in which the precise sequence of events is subject to evolutionary optimization. Coordination of transcription with required fluxes will lead to efficient use of resources. In contrast, the test cases of [44] involve microbial responses to varying environmental conditions and under- and over-expression mutations. Environmental responses must be rapid, flexible, and reversible—criteria a complex, scripted transcriptional response may not satisfy—while transcriptional responses to novel mutations, by definition, cannot have been evolutionarily optimized. This hypothesis could be tested by evaluating performance of the present method on RNA-seq data from mutant maize plants, or plants subject to environmental challenges.

Consistent with this hypothesis, in the present work the use of transcriptomic data (and a limited number of enzyme activity measurements) allowed the correct prediction of a metabolic transition from the base of the leaf to the tip, which could not have been expected based on FBA calculations alone: without such data, all points along the gradient would be identical, and the biomass-production-maximizing solution would be the same at each. The predicted position of the source-sink transition is not perfectly accurate, and the quantitative accuracy of the model cannot be evaluated until the predicted reaction rates are compared to detailed experimental flux measurements, but the results are encouraging and suggest that inference of fluxes from expression data may be more feasible in the specialized context of developmental shifts in metabolism than it is in general.

Potentially further supporting this idea, we note that methods that did not constrain or maximize the growth rate predicted zero growth rates in almost all the test cases studied by Machado and Herrgård [44]. In the present method, the objective function of Eq 3 does not maximize the growth rate, and we have not constrained the growth rate to be nonzero; nonetheless, the method consistently predicts nonzero rates of biomass production (whether a flexible biomass composition is allowed, as above, or the fixed biomass composition is used, as in S7 and S8 Figs).

### Model Reconstruction

Our model is the fourth published genome-scale metabolic reconstruction of the major crop plant *Zea mays*, and the first such reconstruction developed solely from maize data sources, rather than as a direct or indirect adaptation of the *Arabidopsis thaliana* model AraGEM [21].

Direct reaction-to-reaction comparison of iEB5204 with C4GEM [45], iRS1563 [22], and its successor model [46] is difficult because those models use a naming scheme for compounds and reactions ultimately based on KEGG [47, 48] while this model, like its parent database, uses the nomenclature of MetaCyc and the BioCyc database collection. The models are broadly similar in size and biological scope. As published, C4GEM included 1588 reactions associated with 11623 maize genes; iRS1563, 1985 reactions associated with 1563 genes; the model of Simons et al. [46], 3892 unique reactions and 5824 genes; and iEB5204, 2720 reactions with 5204 genes. All models can simulate the production of similar sets of basic biomass constituents (including amino acids, carbohydrates, nucleic acids, lipids and fatty acids, and cell wall components) under photosynthetic and non-photosynthetic conditions and include key reactions of the C4 cycle. The model of Simons et al. [46] also offers extensive coverage of secondary metabolism.

Our computational methods, discussed below, should allow the incorporation of realistic Rubisco kinetics into any of the prior genome-scale models of C4 plant metabolism. However, for the specific goal of integration with transcriptomics data from the leaf developmental gradient, we found it useful to develop the present model, which has several advantages:

**Gene associations** The gene associations included in iEB5204 are those presented in CornCyc [26], which are generated by the PMN Ensemble Enzyme Prediction Pipeline (E2P2) [49], a homology-based protein sequence annotation algorithm trained on a reference dataset of experimentally validated enzyme sequences. The E2P2 approach is more comprehensive and scalable than the development procedures of the previous maize reconstructions (which involve, for example, obtaining gene associations by transferring annotations from *Arabidopsis* genes to their best maize BLAST hits and manually selecting annotations for remaining maize genes from among BLAST hits in other species.) The entire set of gene associations in the FBA model may be readily updated based on improvements in the E2P2 prediction algorithm.
**High-confidence submodel** In developing the fitting algorithm we found that, to obtain plausible metabolic state predictions, a conservative reconstruction was preferable to a comprehensive one. For example, early tests with the comprehensive version of the model suggested that the fitting algorithm often found low-cost solutions involving high fluxes through reactions which, on investigation, we determined were unlikely to be active in maize. Because of the model’s connection to the CornCyc database, it was straightforward to create a reduced, high-confidence version of the model by preferentially excluding reactions not included in any manually curated plant metabolic pathway, even if candidate associated genes had been identified computationally, leading to more realistic results.
**Reproducibility** In an effort to improve the reusability of the model and encourage its application to other data sets, we have provided the full source code (S1 and S2 Protocols) for all calculations presented here, as has been recommended (see, e.g., [50]).

Previous reconstructions do offer two features absent from this model: gene associations for intracellular transport reactions, and gene associations which take into account the structure of protein complexes. Both should be considered in future work.

In agreement with [51], we found that building the model starting from a metabolic pathway database was considerably more straightforward than the standard process of *de novo* reconstruction [52]. Reasonable effort was still required to bring the model to a functional state by identifying reactions or pathways present in the CornCyc database which could not be handled automatically by the Pathway Tools export facility (for example, because they involved polymerization, or could not be checked automatically for conservation violations) and determining how to represent them appropriately in the FBA model.

The model construction process here could readily be adapted to generate metabolic models describing any of the more than 30 crop and model plant species for which Pathway Tools-based metabolic pathway databases [53] have been developed by the Plant Metabolic Network [54], Sol Genomics Network [55], Gramene [56], and others (e.g., [57–59]) allowing the present data-fitting method to be applied to RNA-seq data from those organisms. The level of model development effort required and quality of fit results will vary depending on the extent of curation of the pathway database and quality of the gene function annotations.

### Nonlinear optimization

In contrast to the linear and convex optimization methods employed in nearly all prior constraint-based modeling work, general constrained nonlinear optimization algorithms typically require more effort from the user (who might be required to supply functions which evaluate the first and second derivatives of all constraints with respect to all variables in the problem). They are slower, are more sensitive to choices of starting point and problem formulation, are not guaranteed to converge to an optimal point even if one exists, and, when they do converge to an optimum, cannot guarantee that it is globally optimal.

The software package we present allows the rapid and effective development of metabolic models with nonlinear constraints despite these complications. All necessary derivatives of constraint functions are taken analytically, and Python code to evaluate them is automatically generated. A model in SBML format may be imported, nonlinear constraints added and removed, and the problem repeatedly solved to test various design choices, solver options, and initial points, all within an interactive session, with a minimum of initial investment of effort in programming.

In the present case, agreement between nonlinear FBA calculations that maximized CO_2_ assimilation and the predictions of classical physiological models confirmed that the true, globally optimal CO_2_ assimilation rate was found successfully. For the data-fitting calculations, where the true optimal cost is not known, we cannot exclude the possibility that there exist other optimal solutions, qualitatively distinct from the flux distributions and quasi-optimal regions presented above, with equivalent or lower costs. In practice, we encountered occasional cases in which reaction or pathway fluxes were initially predicted to be zero even when associated with nonzero data, despite the existence of a superior alternative solution with nonzero predicted fluxes. A step to detect and correct these situations was incorporated into the fitting algorithm.

Many future applications for the software are possible. Our approach to Rubisco kinetics may easily be extended to other models of C4 metabolism or, more generally, to any FBA calculation in a photosynthetic organism where the CO_2_ level at the Rubisco active site, and thus the Rubisco oxygenation/carboxylation ratio, is not known *a priori*. A published genome-scale metabolic reconstruction of the model alga *Chlamydomonas reinhardtii*, for example, was identified by the authors as being deficient in describing algal metabolism under low CO_2_ conditions due to the fact that the Rubisco carboxylase and oxygenase fluxes were treated as independent and not (as we have done here) competitive [60].

Ensuring that rates of Rubisco oxygenation, Rubisco carboxylation, and PEPC carboxylation are consistent with our knowledge of their kinetics is a special case of the more general problem of integrating kinetic and constraint-based modeling. Diverse approaches to this issue have been extensively developed, including dynamic FBA [61], k-OptForce [62], genome-scale kinetic modeling [63, 64], and others (e.g., [65–67]). To our knowledge, no prior work has simply imposed kinetic laws as additional, nonlinear constraints in the ordinary FBA optimization problem. Our results demonstrate the potential of this approach in systems where the kinetics of a few well-understood reactions are crucial. It remains to be seen how many kinetic laws may be incorporated in this way at once, and to what extent their introduction usefully constrains the space of possible steady-state flux distributions even when relevant kinetic parameters are not known (but instead are treated as optimizable variables, an approach with connections to ensemble kinetic modeling [68]).

Nonlinear constraints may also be of use in enforcing thermodynamic realizability of flux distributions, and relaxing requirements of linearity or convexity may stimulate the development of novel objective functions—either for data integration purposes, as here, or as alternatives to growth-rate maximization.

### The whole-leaf model

Large-scale metabolic models of interacting cells of multiple types first appeared in 2010, with C4GEM [45] and a model of human neurons interacting with their surrounding astrocytes [69]. Many more complex multicellular FBA models have since appeared, including studies of the metabolism of interacting communities of microbial species in diverse natural environments or artificial co-cultures [70–76] (also [77] at a smaller scale) and of the metabolic capacities of host animals and their symbionts [78] or parasites [79]. In plants, diurnal variation in C3 and CAM plant metabolism has been simulated with a model which represents different phases of the diurnal cycle with different abstract compartments, with transport reactions representing accumulation of metabolites over time [80].

In the most direct antecedent of the present work, Grafahrend-Belau and coauthors developed a multiscale model of barley metabolism [81] which represented leaf, stem, and seed organs as subcompartments of a whole-plant FBA model, with nutrients exchanged through the phloem. Combining the FBA model with a high-level dynamic model of plant metabolism allowed them to predict changes in metabolism over time, including the transition between a biomass-producing sink state and a fructan-remobilizing source state in the stem late in the plant’s life cycle.

The whole-leaf model presented here occupies an intermediate position between prior C4 models, with single mesophyll and bundle sheath cells, and multi-organ whole-plant models such as [81]. It represents the first attempt to model spatial variations in metabolic state within a single organ, allowing the study of developmental transitions in leaf metabolism by incorporating data from more and less differentiated cells at a single point in time, rather than modeling development dynamically.

Other interacting cell models incorporate *a priori* qualitative differences in the metabolic capabilities of their components (e.g., leaf, stem, and seed, or neurons and astrocytes). In contrast in the work presented here, in order to allow the metabolic differences between any two adjacent points to be purely quantitative, the same metabolic network must be used for all points. This simplifies the process of model creation but implies that meaningful predictions of spatial variation depend entirely on the integration of (spatially resolved) experimental data. The ability of the model to capture the experimentally observed shift from sink to source tissue along the developmental gradient based on RNA-seq and enzyme activity measurements shows that this may be done successfully with high-resolution -omics data and careful model construction.

## Methods

### Reconstruction process

A local copy of CornCyc 4.0 [26] was obtained from the Plant Metabolic Network and a draft metabolic model was created using the MetaFlux module of Pathway Tools 17.0 [51]. The resulting model, including reaction reversibility information, was converted to SBML format and iteratively revised, as described in detail in S1 Appendix, until all desired biomass components could be produced under both heterotrophic and photosynthetic conditions and realistic mitochondrial respiration and photorespiration could operate.

An overall biomass reaction was adapted from iRS1563 [22] with minor modifications to components and stoichiometry, as detailed in S1 Appendix. To allow calculations with flexible biomass composition, individual sink reactions were added for most species participating in the biomass reaction, as well as several relevant species (including chlorophyll) not originally included in the iRS1563 biomass equation, for which synthesis pathways were identified in CornCyc.

Core metabolic pathways were assigned appropriately to subcellular compartments (e.g., the TCA cycle and mitochondrial electron transport chain to the mitochondrion; the light reactions of photosynthesis, the Calvin cycle, and some reactions of the C4 cycle to the chloroplast; and some reactions of the photorespiratory pathway to the peroxisome) and the intracellular transport reactions necessary for their operation were added.

The model was thoroughly tested for consistency and conservation violations, confirming that no species could be created without net mass input or destroyed without net mass output (except species representing light, which can be consumed to drive futile cycles.)

The base metabolic model iEB5204 is provided in SBML format as S1 Model. Gene association rules for reactions with associated genes in CornCyc are provided following COBRA conventions [82]. Additional annotations give the record in the CornCyc database associated with each reaction and species, where applicable.

To produce the higher-confidence version of the reconstruction, iEB2140 (S2 Model), reactions in the base model which were not associated with any identified metabolic pathway in CornCyc, and those for which no genes for a catalyzing enzyme had been identified by computational function prediction, were removed from the model if their removal did not prevent photosynthesis, photorespiration, or the production of any biomass component. Then, all reactions which could not achieve nonzero steady-state rates were removed.

### Mesophyll-bundle sheath model

A model for leaf tissue (S3 Model) was created by taking two copies of the high-confidence model, representing mesophyll and bundle sheath cells, and adding reactions representing transport through the plasmodesmata which connect the cytoplasmic spaces of adjacent cells. For details, see S2 Appendix.

### Physiological constraints

Rubisco carboxylase and oxygenase rates *v*_*c*_ and *v*_*o*_ in mesophyll and bundle sheath chloroplasts were constrained to obey Michaelis-Menten kinetic laws with competitive inhibition,

$$\begin{array}{c} \begin{array}{cc} v_{c} & =\frac{v_{c,\text{max}}\left[{\text{CO}}_{2}\right]}{\left[{\text{CO}}_{2}\right]+k_{c}\left(1+\frac{\left[{\text{O}}_{2}\right]}{k_{o}}\right)}\\ v_{o} & =\frac{v_{o,\text{max}}\left[{\text{O}}_{2}\right]}{\left[{\text{O}}_{2}\right]+k_{o}\left(1+\frac{\left[{\text{CO}}_{2}\right]}{k_{c}}\right)}, \end{array} \end{array}$$ (5)

and the relationship *v*_*o*, max_/*v*_*c*, max_ = *k*_*O*_/(*k*_*C*_ ⋅ *S*_*R*_) was imposed, from which Eq (2) follows [15]. The Michaelis-Menten constants for oxygen and carbon dioxide *k*_*C*_ and *k*_*O*_ and the Rubisco specificity *S*_*R*_ were set to values typical of C4 species: *k*_*C*_, 650 μmol mol^-1^; *k*_*O*_, 450 mmol mol^-1^; *S*_*R*_, 2590 [15].

The rate of PEP carboxylation in the mesophyll was governed by an appropriate kinetic law,

$$\begin{array}{c} v_{p}=\frac{v_{p,\text{max}}\left[{\text{CO}}_{2}\right]}{k_{C,p}+\left[{\text{CO}}_{2}\right]} \end{array}$$ (6)

with an appropriate *k*_*C*,*p*_ (80mmol mol^-1^, [15]).

The parameters *v*_*p*max_ and *v*_*c*,max_ representing the total amount of Rubisco and PEPC available may be fixed to permit comparison to models parameterized in those terms or allowed to vary.

Rates of oxygen and carbon dioxide diffusion from the bundle sheath to the mesophyll, *L* and *L*_*O*_, were constrained to obey the relationship

$$\begin{array}{c} \begin{array}{cc} L & =g_{s}\left({\text{CO}}_{2,BS}-{\text{CO}}_{2,ME}\right)\\ L_{O} & =g_{s,O}\left({\text{O}}_{2,BS}-{\text{O}}_{2,ME}\right) \end{array} \end{array}$$ (7)

with the bundle sheath oxygen conductance *g*_*s*, *O*_ set to 0.047*g*_*s*_, where *g*_*s*_ is the bundle sheath CO_2_ conductance [15]. All simulations used the bundle sheath CO_2_ conductance measured by [36] for maize plants grown under high light, 1.03±0.18 μmol m^-2^ s^-1^. While *g*_*s*_ undoubtedly varies along the developmental gradient, its deviation from this value (measured in fully-expanded leaves, 3-4 weeks after planting) is likely greatest below the region of high suberin synthesis identified 4 cm from the leaf base [31]; as the C4 cycle was not predicted to operate at high rates in this region, the impact of this discrepancy should be limited.

Resistance to CO_2_ diffusion from the intercellular airspace to the mesophyll cells was neglected; ref. [83] reported the relevant conductance was approximately 1 mmol m^-2^ s^-1^ in maize under a variety of conditions, suggesting the mesophyll and intercellular CO_2_ levels would differ only slightly at the rates of CO_2_ assimilation and release dealt with here. Similarly, all intracellular compartments were taken to have equal CO_2_ concentrations.

### Optimization calculations

The nonlinear modeling package uses the libsbml python bindings to read SBML files [84] and an internal representation of SBML models derived from the SloppyCell package [85, 86]. IPOPT calculations used version 3.11.8 with the linear solver ma97 from the HSL Mathematical Software Library [87]. Where not specified, convergence tolerance was 10^−5^, or 10^−4^ in FVA calculations. To solve purely linear problems (e.g., to test the production of biomass species during the reconstruction process, where nonlinear constraints were not used) the GNU Linear Programming Kit, version 4.47 [88], was called through a Python interface [89].

### Comparison with other models

Python code used to calculate the predictions of the models of von Caemmerer [15] for comparison with nonlinear optimization results is provided in S2 Protocol.

### Integrating biochemical and RNA-seq data

#### RNA-seq datasets

To obtain mesophyll- and bundle-sheath-specific expression levels at 15 points, we combined the non-tissue-type-specific data of Wang et al. [31], measured at 1-cm spatial resolution, with the tissue-specific data of Tausta et al. [32] obtained by using laser capture microdissection (LCM)—measured 4 cm, 8 cm and 13 cm from the leaf base (the upper three highlighted positions in Fig 3b), as explained in S2 Appendix.

#### Enzyme activity measurements

The full list of reaction rates constrained by enzyme activity measurements from [31] is given in S2 Appendix.

#### Handling reversible reactions

The objective function (Eq (3)) optimizes the agreement between the absolute value of the flux through each reaction with its data. The resulting optimization problem cannot be solved directly with the methods used here because the absolute value function is not continuously differentiable. To circumvent this limitation, directions for reactions considered reversible (based on information from CornCyc [26]) were determined in a heuristic method similar in spirit to that of [33], detailed in S2 Appendix.

## Supporting Information

S1 Fig Phloem transport. Transport of nitrogen (upper panel) and sulfur (lower panel) through the phloem in the best-fitting solution. Dotted lines indicate minimum and maximum predicted values consistent with an objective function value no more than 0.1% worse than the optimum.
(PDF)

S2 Fig Photosystem II in mesophyll and bundle sheath. Dashed and dotted lines indicate minimum and maximum predicted values consistent with an objective function value no more than 0.1% worse than the optimum.
(PDF)

S3 Fig Bundle sheath PEPC flux in the best-fitting solution. Dotted lines indicate minimum and maximum predicted values consistent with an objective function value no more than 0.1% worse than the optimum.
(PDF)

S4 Fig Summary of predictions for the gradient model using the least-squares method without per-reaction scale factors. In Eq (3), *s*_*i*_ = 0 for all reactions *i*. (a) Sucrose and CO_2_ uptake rates (compare to Fig 3a). (b) Rates of carboxylation by PEPC and Rubisco (compare to Fig 4b). (c) Predicted rate for the reactions of the chlorophyllide A synthesis pathway (compare to Fig 6b). (d) Predicted rates at the arogenate branch point (compare to Fig 6d). (e) Predicted oxygen and carbon dioxide levels in the bundle sheath, with straight lines showing mesophyll levels (compare to Fig 4d). (f) Distribution of correlation coefficients between data and predicted fluxes for each reaction. (blue, this method; red, standard method.) Correlation coefficients for reactions with zero predicted flux are taken to be zero, resulting in the visible peak in the histogram.
(PDF)

S5 Fig Summary of predictions for the gradient model using the E-Flux method. For explanation of each panel, see S4 Fig.
(PDF)

S6 Fig Summary of predictions for the gradient model using the E-Flux method with fixed biomass composition. The biomass composition is fixed to that used by iRS1563, as adapted (see S1 Appendix). For explanation of each panel, see S4 Fig. Note that the chlorophyllide A synthesis pathway is blocked when the fixed biomass composition is used.
(PDF)

S7 Fig Summary of predictions for the gradient model with fixed biomass composition. For explanation of each panel, see S4 Fig. Note that the chlorophyllide A synthesis pathway is blocked when the fixed biomass composition is used.
(PDF)

S8 Fig Predicted biomass production rates in mesophyll and bundle sheath cells with fixed biomass composition. (PDF)

S9 Fig Predicted variable values in an FBA calculation that does not incorporate expression data, compared to the best-fit and E-Flux methods. The FBA calculation minimizes total flux while achieving the same total rate of CO_2_ assimilation as predicted at the tip of the leaf in the fitting results. Left panel, FBA reaction rates vs. reaction rates predicted at the tip of the leaf in the best-fitting solution; right panel, FBA reaction rates vs. reaction rates predicted at the tip of the leaf by the E-Flux method. Axis limits exclude a small number of reactions of particularly large flux. Fluxes in μmol m^-2^ s^-1^.
(PDF)

S10 Fig Summary of predictions for the gradient model, omitting nonlinear kinetic law constraints. Effects of relaxing the requirement that predicted PEPC, Rubisco, and oxygen and carbon dioxide obey the kinetic laws of Eqs (5), (6) and (7). For details, see S2 Appendix. (a) Sucrose and CO_2_ uptake rates (compare to Fig 3a). (b) Rates of carboxylation by PEPC and Rubisco. PEPC activity increases more uniformly along the gradient, compared to the results shown in Fig 4a. (c) Predicted rates of bundle sheath decarboxylation reactions, showing increased PEPCK activity compared to the results shown in Fig 4b. (d) Predicted rates of oxygenation by Rubisco in the bundle sheath, with and without nonlinear kinetic laws. (e) Predicted rates of diffusion of carbon dioxide from bundle sheath to mesophyll, with and without nonlinear kinetic laws. (f) Cumulative histogram of correlation coefficients for fluxes of each reaction along the leaf gradient, predicted with and without nonlinear kinetic laws.
(PDF)

S11 Fig Predicted rates of production of selected subcategories of biomass components along the leaf gradient, illustrating the model’s capability to simulate variations in biomass composition. (a) Predicted production of cellulose, amino acids, nucleic acids, and lipids and fatty acids all show a pronounced peak at the base of the leaf and are higher in the predicted heterotrophic source region, consistent with the interpretation of this region as an area of active cell growth and division. (b) In contrast, predicted chlorophyll production is relatively steady along the leaf, while ascorbate production increases from the source-sink transition to the tip of the leaf.
(PDF)

S1 Appendix Details of the metabolic model development process. (PDF)

S2 Appendix Implementation details. (PDF)

S3 Appendix Information on the model’s two alternative sets of biomass-producing reactions, and related reactions and constraints. (TXT)

S1 Table Detailed parameters contributing to the effective PEP regeneration rate: reactions in the genome-scale model which contribute to the effective maximum PEP regeneration capacity, and the number of genes associated with each. In addition to the reactions listed, transport capacities of pyruvate, PEP, alanine, aspartate and malate across the plasmodesmata and pyruvate, PEP, malate and oxaloacetate across the chloroplast inner membrane could limit this rate; the model currently associates no genes with these transport reactions.
(PDF)

S2 Table Predicted variable values along the leaf gradient. To assess the precision with which the model predicts the value of any variable requires a separate optimization calculation, which has been done only for the subset of variables for which upper and lower bounds are given in S3 Table below; thus the appropriate number of significant figures to which these values should be reported is not clear, but will generally be fewer than have been given here. These predictions were made using the set of biomass reactions that allows flexible biomass composition; the set of biomass reactions corresponding to a fixed biomass composition thus have zero fluxes. See S3 Appendix for further details.
(TXT)

S3 Table Upper and lower bounds on predicted values of selected variables along the leaf gradient, from FVA calculations. (TXT)

S4 Table Input data for the flux prediction calculations. Sheet 1, RNA-seq data (FPKM) from the experiments of Wang et al [31] (nonconsecutive segment order present in original.) Sheet 2, RNA-seq data (in RPKM) from the experiments of Tausta et al [32]. Sheet 3, cell-type-specific expression estimates (in FPKM) obtained by combining the data of sheets 1 and 2 as described in section 3 of S2 Appendix. Sheet 4, estimated standard deviations (in FPKM) for the expression estimates of sheet 3, obtained as described in section 3 of S2 Appendix. Sheet 5, data associated with reactions in the model by combining the data from their associated genes in sheet 3 and rescaling, as described in section 3 of S2 Appendix. (These are the values *d*_*ij*_ in Eq 3). Note in some cases this data is not associated with a reaction rate, but instead a parameter in a kinetic law constraint (for example, expression data for PEP carboxylase in the mesophyll is associated with ms_active_pepc, the model’s internal term for *v*_*p*,max_ of Eq 6). Sheet 6, standard deviations associated with the data of sheet 3, obtained from the standard deviations in the expression estimates of genes associated with each reaction (sheet 4) as described in section 3 of S2 Appendix. (These are the values *δ*_*ij*_ in Eq 3). Sheet 7, enzyme activity data from Wang et al [31], rescaled as described in section 4 of S2 Appendix. Units are micromole per second per square meter of leaf surface area. These are the values *E*_*jk*_ in Eq 4. Sheet 8, table of reactions in the model constrained by the activity data for each enzyme. Note that in some cases reaction rates are not constrained directly; instead, the constraint is applied to parameters in kinetic law constraints. For example, data for rubisco is used to constrain the sum of ms_active_rubisco and bs_active_rubisco, the model’s internal variables corresponding to *v*_*c*,max_ in Eq 5 in mesophyll and bundle sheath compartments.
(XLSX)

S1 Model iEB5204 in SBML format. (XML)

S2 Model iEB2140 in SBML format. (XML)

S3 Model iEB2140x2 in SBML format. (XML)

S1 Protocol Source code for the nonlinear constraint-based modeling package fluxtools. (GZ)

S2 Protocol Source code and input files for the calculations discussed above. (GZ)
