# Supplementary figures and images for: Multiscale Metabolic Modeling of C4 Plants: Connecting Nonlinear Genome-Scale Models to Leaf-Scale Metabolism in Developing Maize Leaves
Source: PLoS One. 2016 Mar 18;11(3):e0151722. doi: 10.1371/journal.pone.0151722 (PMC4807923; doi:10.1371/journal.pone.0151722)

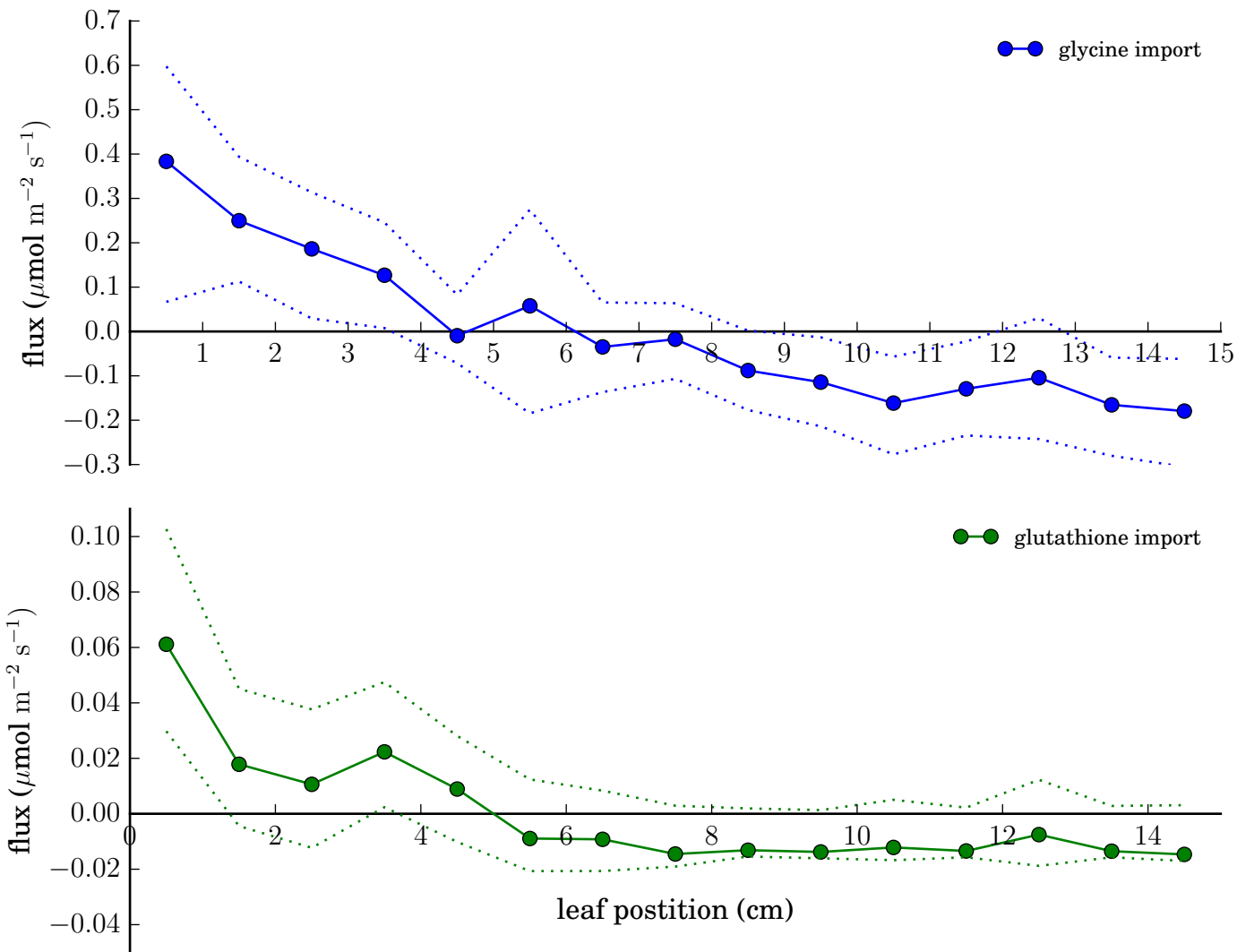

Supplement: S1 Fig — Transport of nitrogen (upper panel) and sulfur (lower panel) through the phloem in the best-fitting solution. Dotted lines indicate minimum and maximum predicted values consistent with an objective function value no more than 0.1% worse than the optimum. (PDF) [file pone.0151722.s001.pdf]

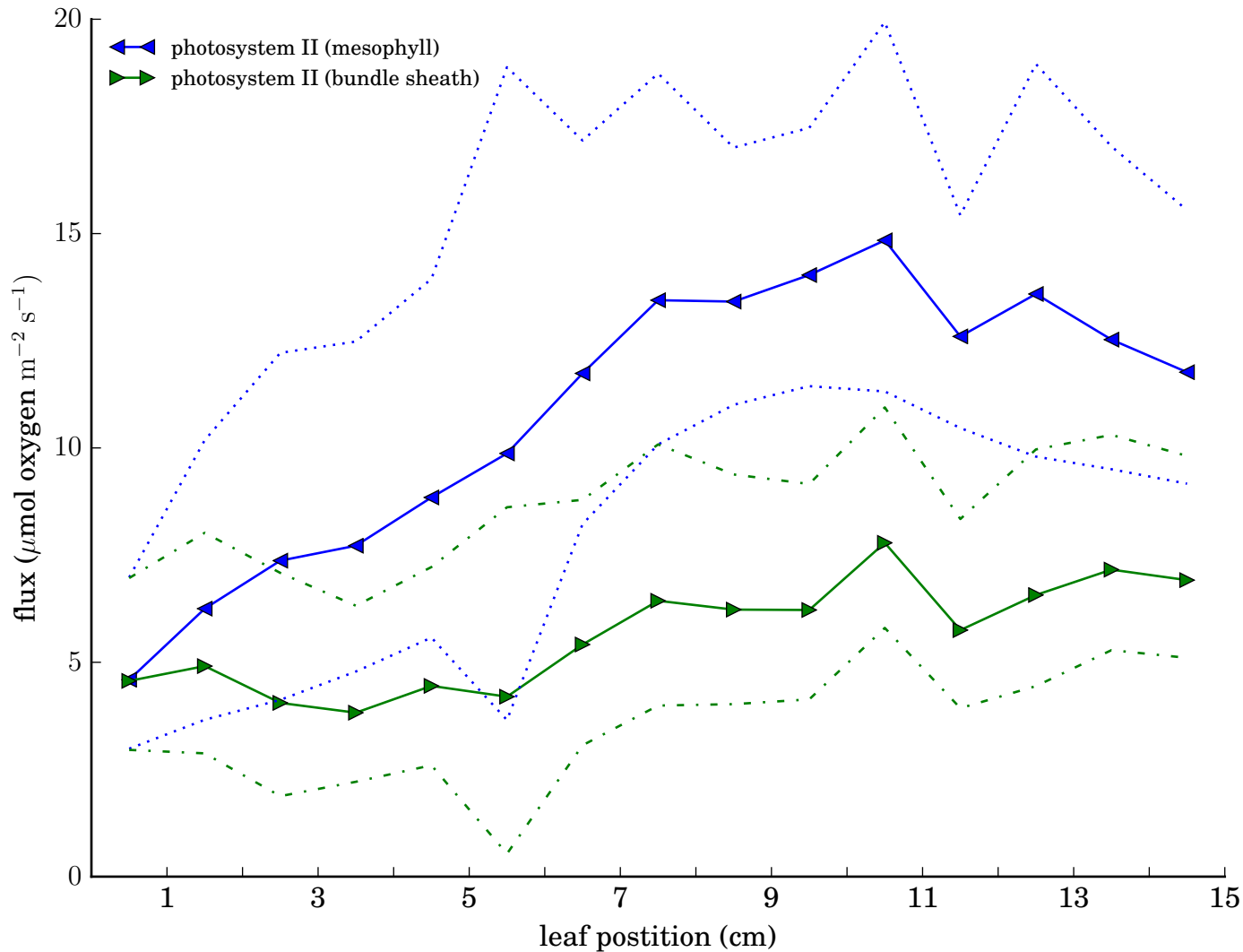

Supplement: S2 Fig — Dashed and dotted lines indicate minimum and maximum predicted values consistent with an objective function value no more than 0.1% worse than the optimum. (PDF) [file pone.0151722.s002.pdf]

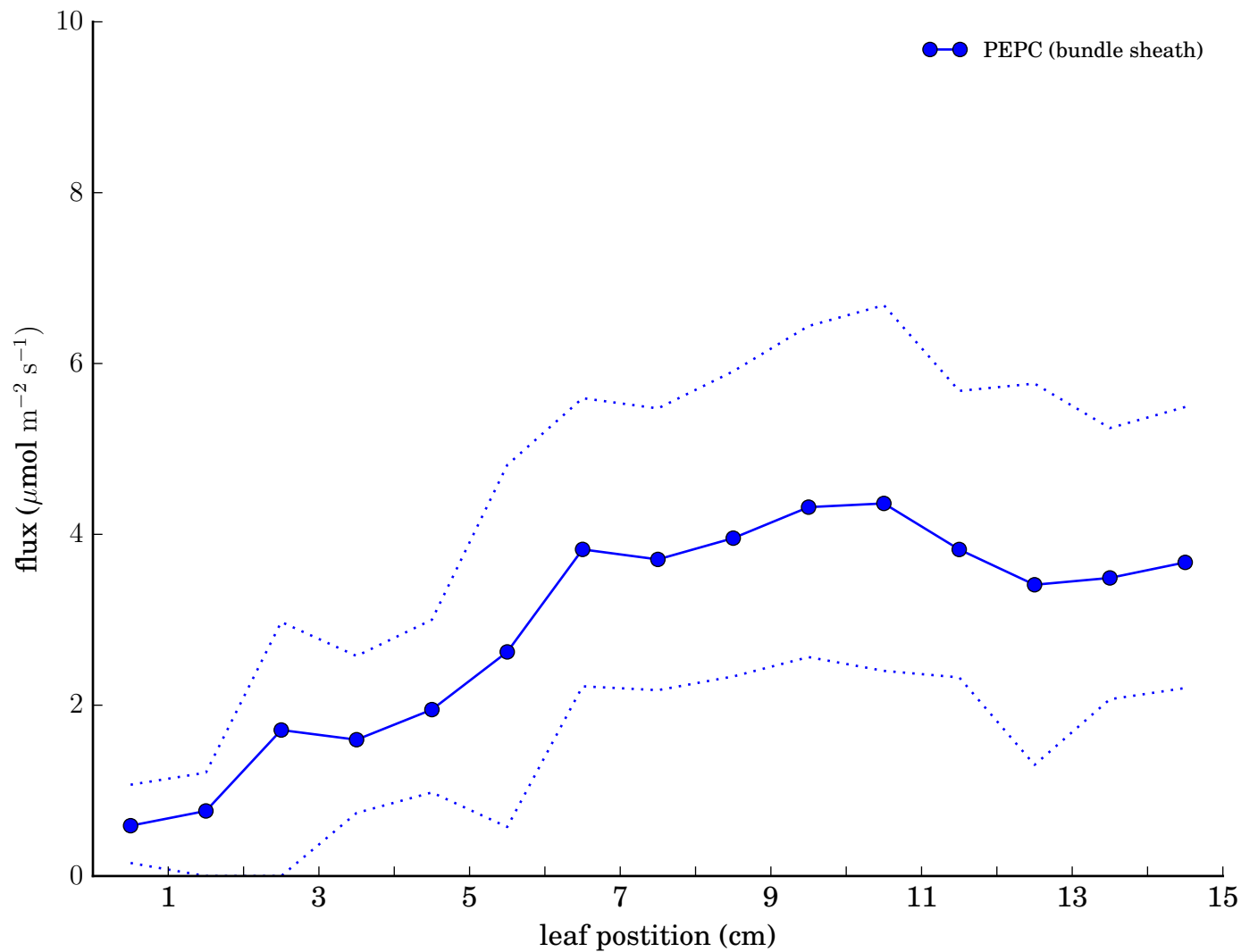

Supplement: S3 Fig — Dotted lines indicate minimum and maximum predicted values consistent with an objective function value no more than 0.1% worse than the optimum. (PDF) [file pone.0151722.s003.pdf]

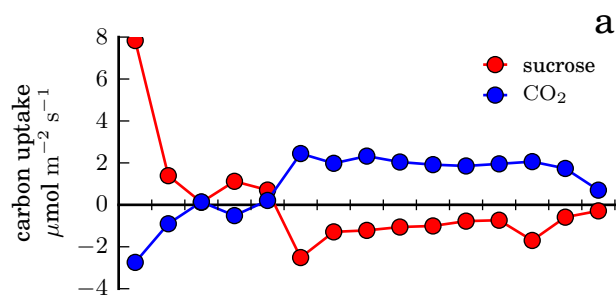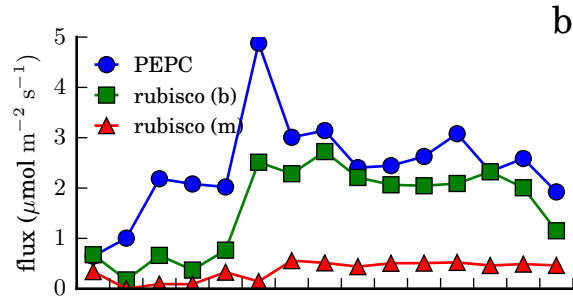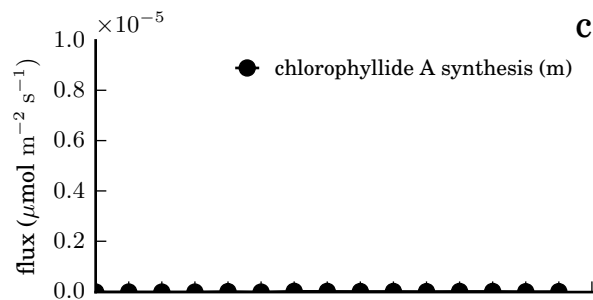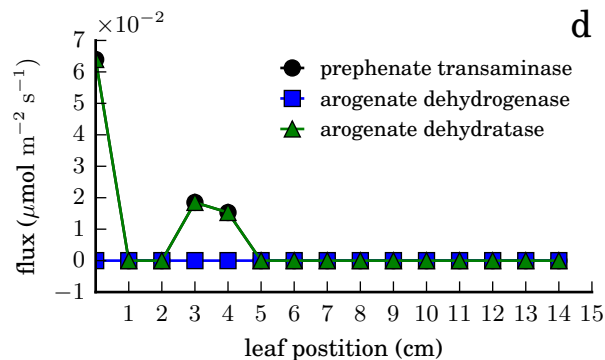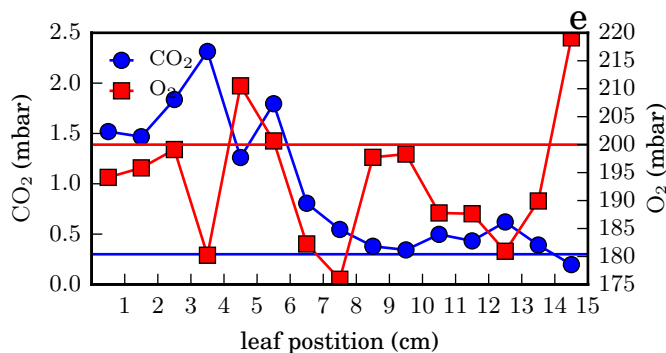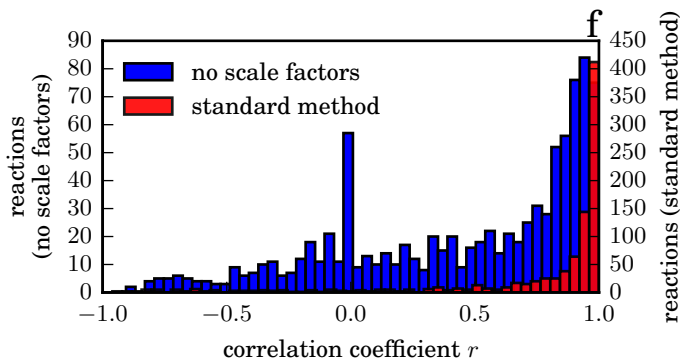

Supplement: S4 Fig — In Eq (3), si = 0 for all reactions i. (a) Sucrose and CO2 uptake rates (compare to Fig 3a). (b) Rates of carboxylation by PEPC and Rubisco (compare to Fig 4b). (c) Predicted rate for the reactions of the chlorophyllide A synthesis pathway (compare to Fig 6b). (d) Predicted rates at the arogenate branch point (compare to Fig 6d). (e) Predicted oxygen and carbon dioxide levels in the bundle sheath, with straight lines showing mesophyll levels (compare to Fig 4d). (f) Distribution of correlation coefficients between data and predicted fluxes for each reaction. (blue, this method; red, standard method.) Correlation coefficients for reactions with zero predicted flux are taken to be zero, resulting in the visible peak in the histogram. (PDF) [file pone.0151722.s004.pdf]

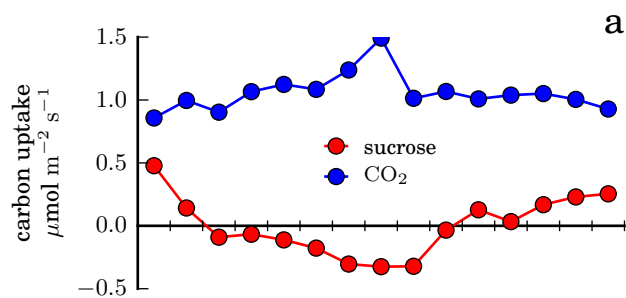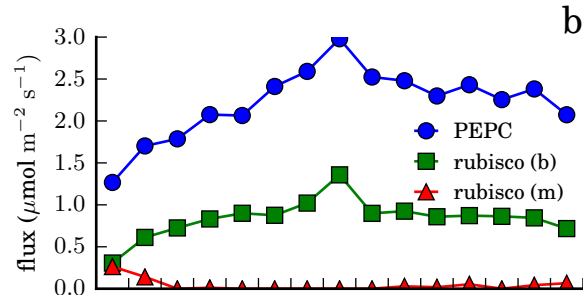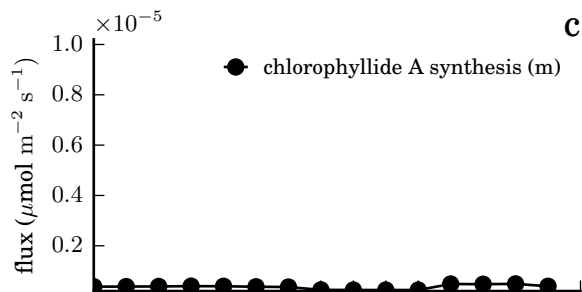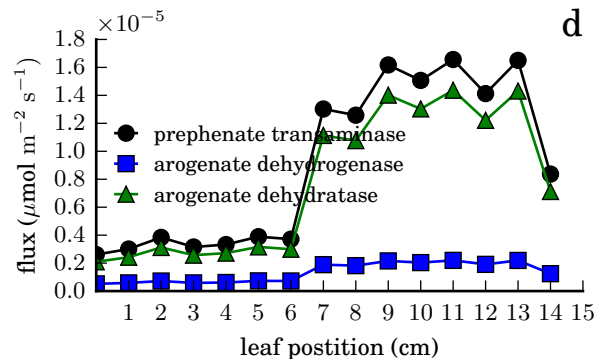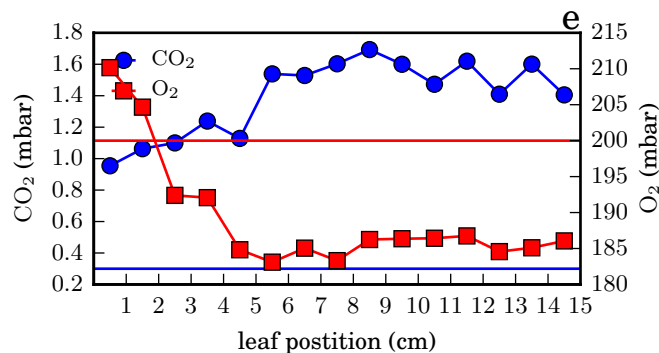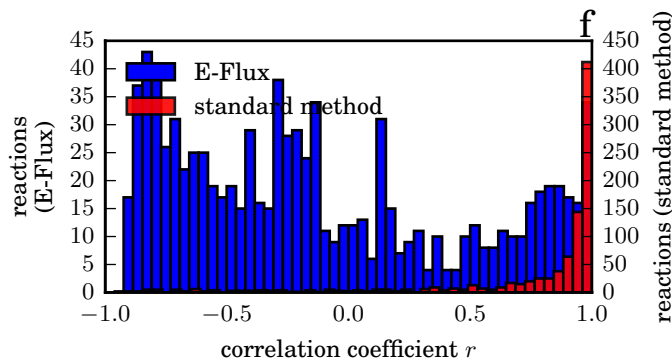

Supplement: S5 Fig — For explanation of each panel, see S4 Fig. (PDF) [file pone.0151722.s005.pdf]

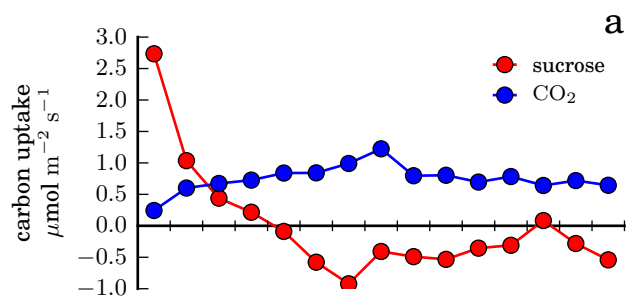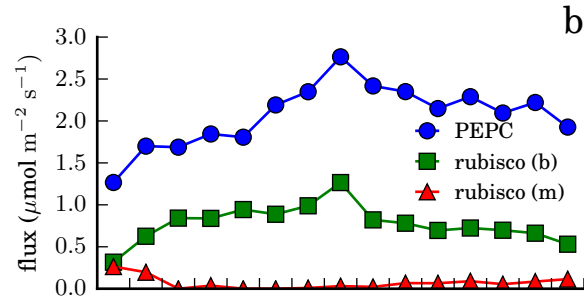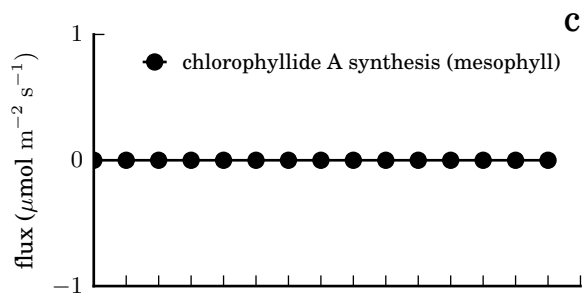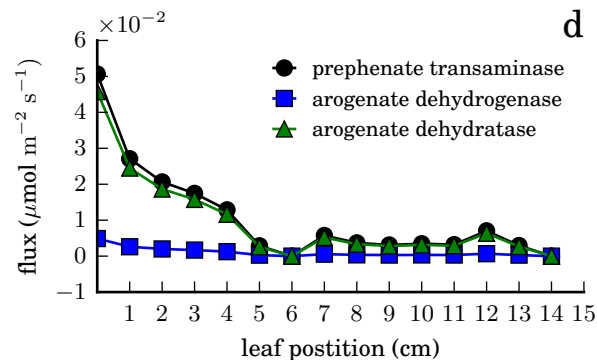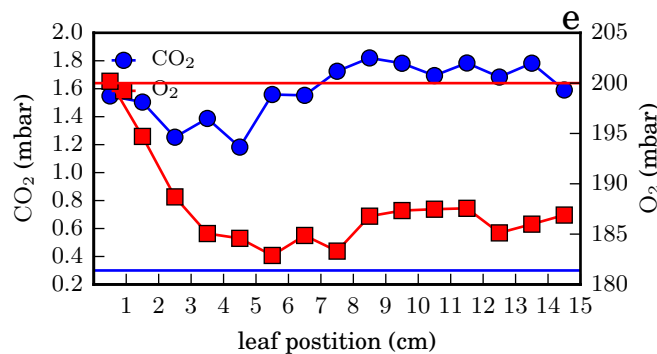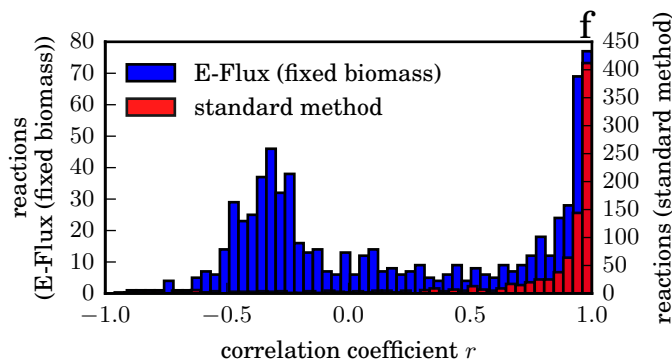

Supplement: S6 Fig — The biomass composition is fixed to that used by iRS1563, as adapted (see S1 Appendix). For explanation of each panel, see S4 Fig. Note that the chlorophyllide A synthesis pathway is blocked when the fixed biomass composition is used. (PDF) [file pone.0151722.s006.pdf]

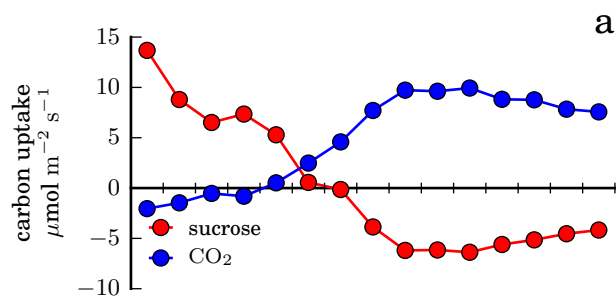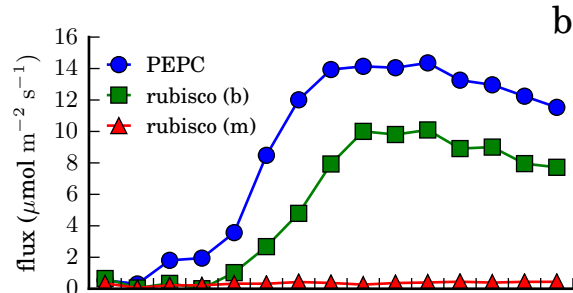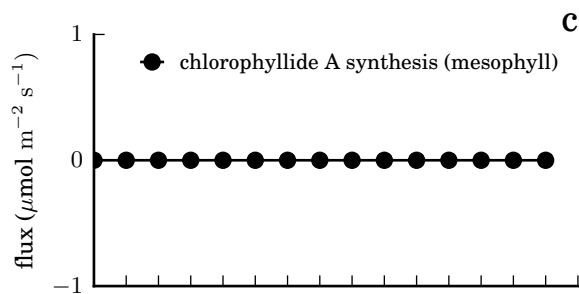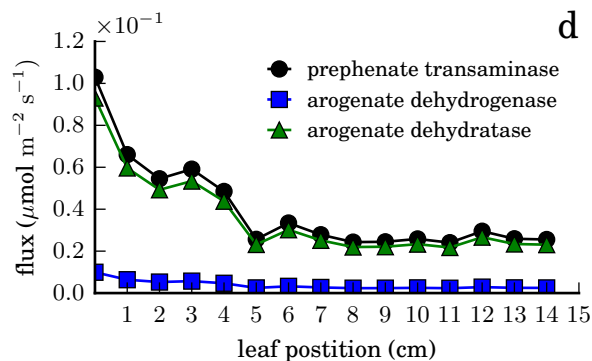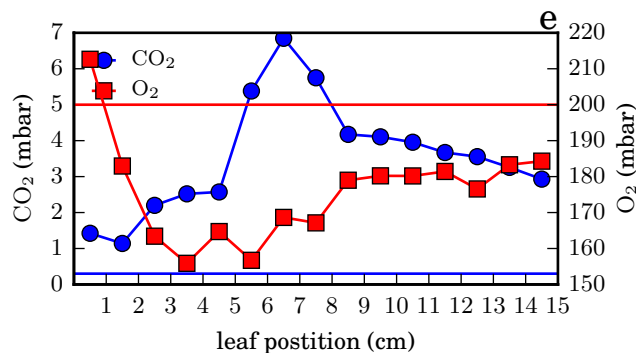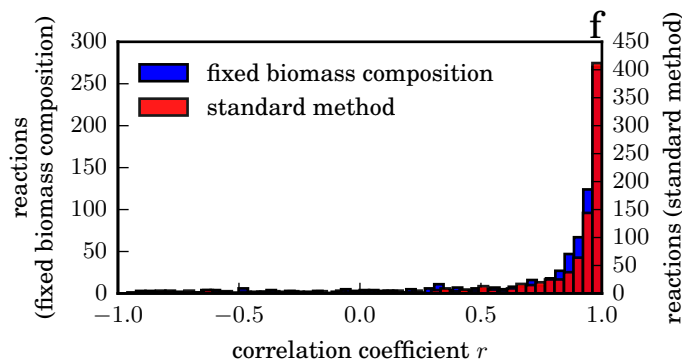

Supplement: S7 Fig — For explanation of each panel, see S4 Fig. Note that the chlorophyllide A synthesis pathway is blocked when the fixed biomass composition is used. (PDF) [file pone.0151722.s007.pdf]

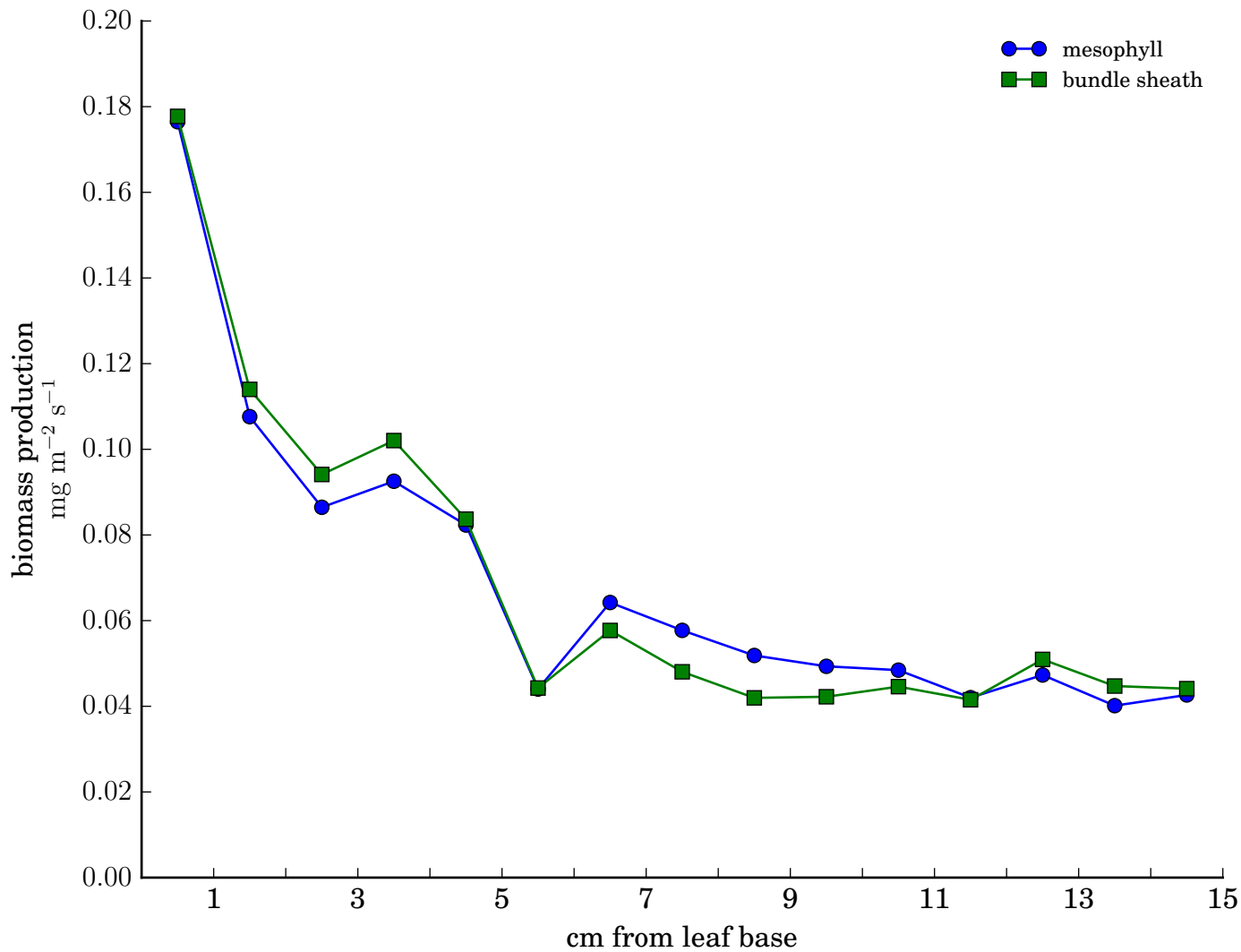

Supplement: S8 Fig — (PDF) [file pone.0151722.s008.pdf]

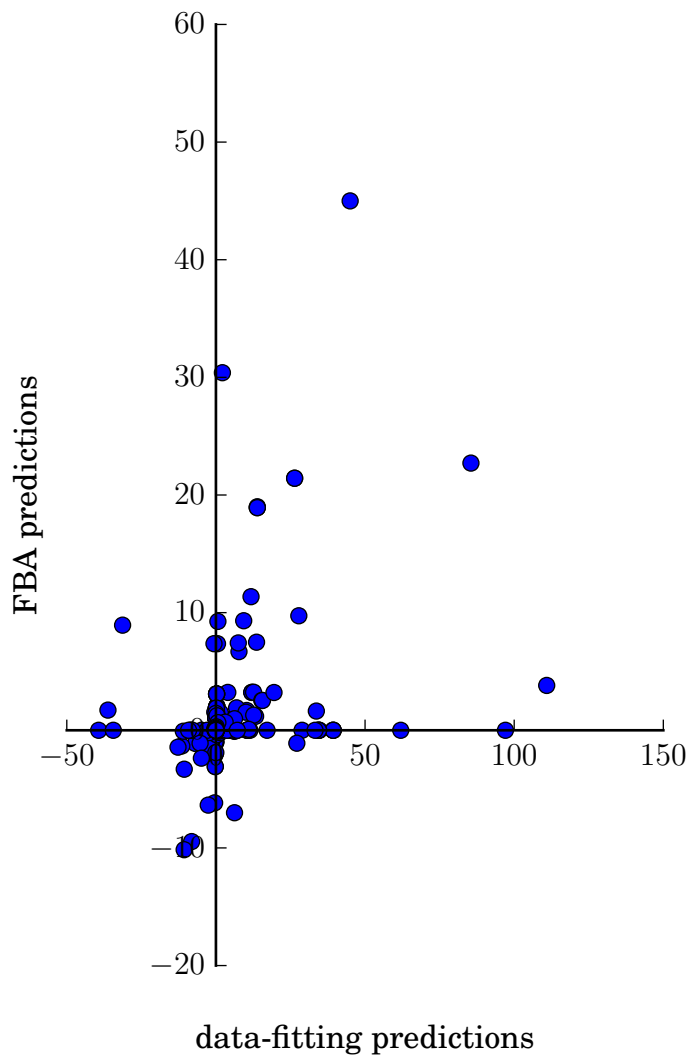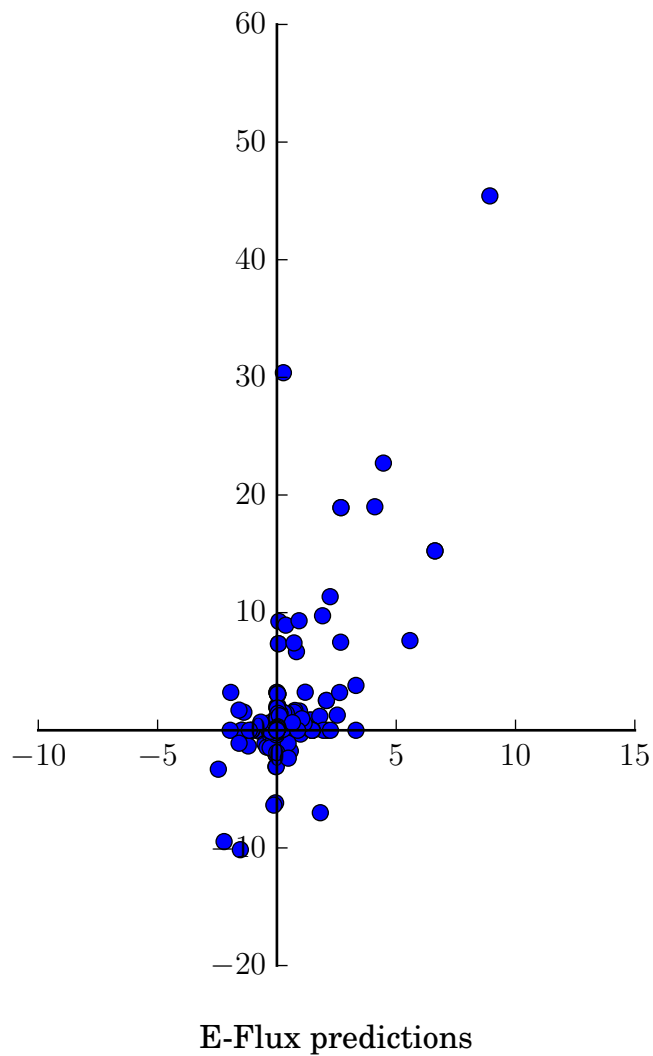

Supplement: S9 Fig — The FBA calculation minimizes total flux while achieving the same total rate of CO2 assimilation as predicted at the tip of the leaf in the fitting results. Left panel, FBA reaction rates vs. reaction rates predicted at the tip of the leaf in the best-fitting solution; right panel, FBA reaction rates vs. reaction rates predicted at the tip of the leaf by the E-Flux method. Axis limits exclude a small number of reactions of particularly large flux. Fluxes in μmol m-2 s-1. (PDF) [file pone.0151722.s009.pdf]

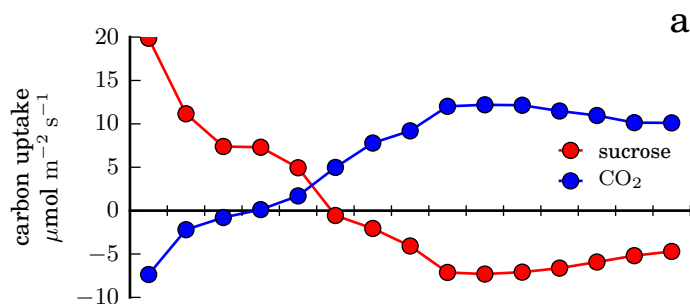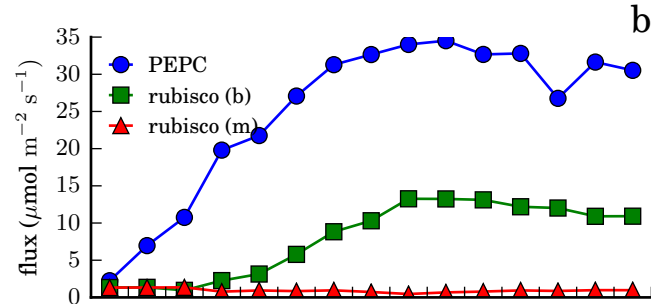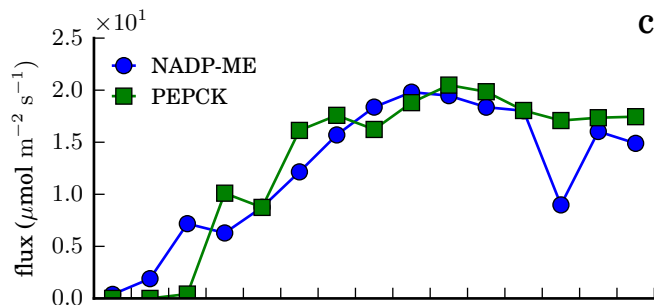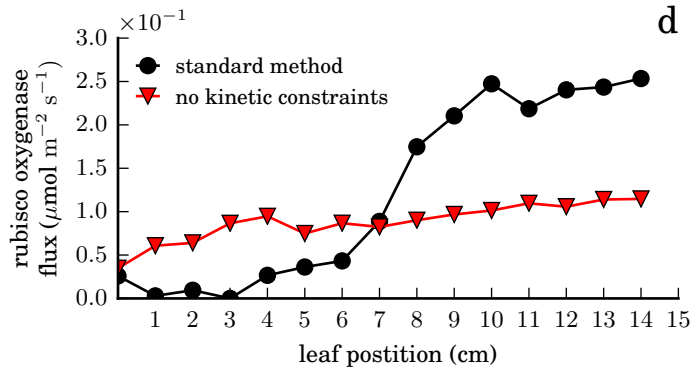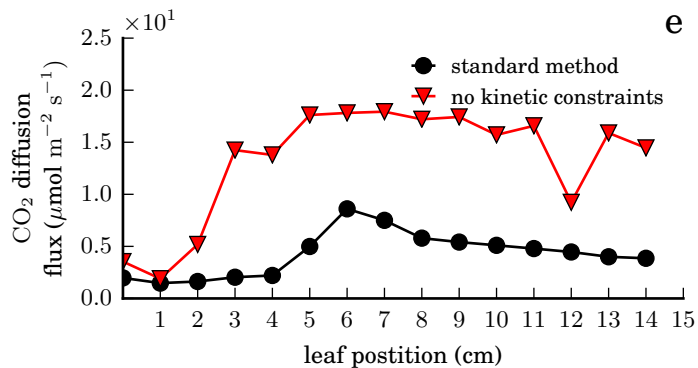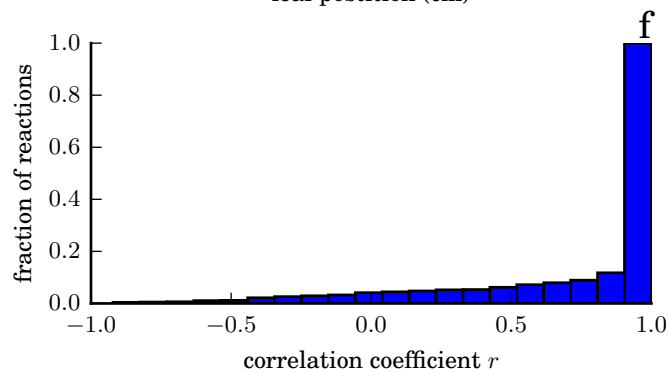

Supplement: S10 Fig — Effects of relaxing the requirement that predicted PEPC, Rubisco, and oxygen and carbon dioxide obey the kinetic laws of Eqs (5), (6) and (7). For details, see S2 Appendix. (a) Sucrose and CO2 uptake rates (compare to Fig 3a). (b) Rates of carboxylation by PEPC and Rubisco. PEPC activity increases more uniformly along the gradient, compared to the results shown in Fig 4a. (c) Predicted rates of bundle sheath decarboxylation reactions, showing increased PEPCK activity compared to the results shown in Fig 4b. (d) Predicted rates of oxygenation by Rubisco in the bundle sheath, with and without nonlinear kinetic laws. (e) Predicted rates of diffusion of carbon dioxide from bundle sheath to mesophyll, with and without nonlinear kinetic laws. (f) Cumulative histogram of correlation coefficients for fluxes of each reaction along the leaf gradient, predicted with and without nonlinear kinetic laws. (PDF) [file pone.0151722.s010.pdf]

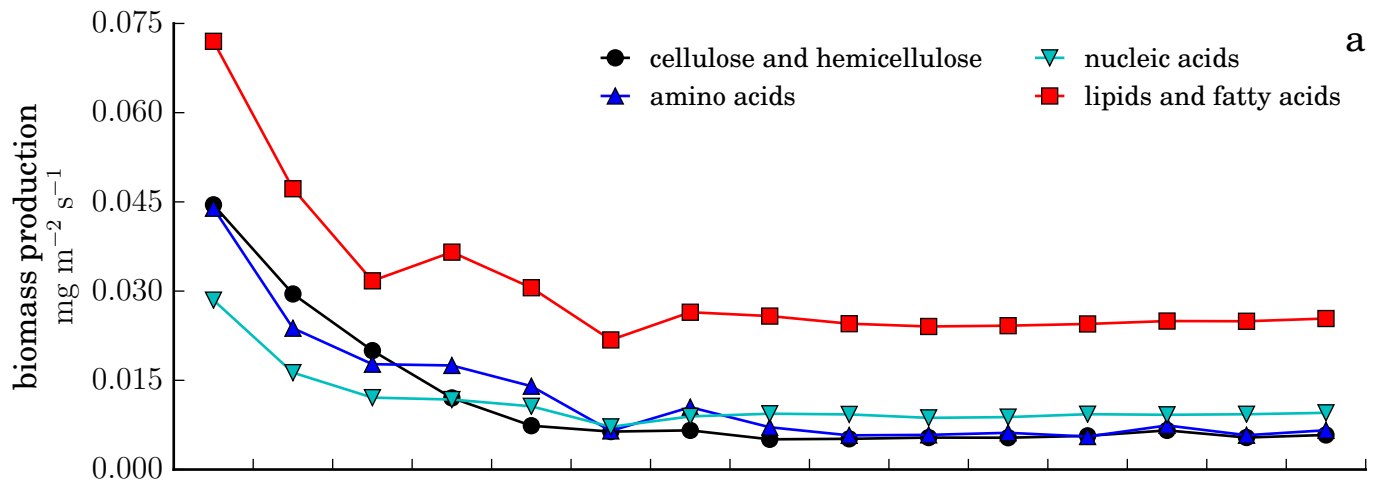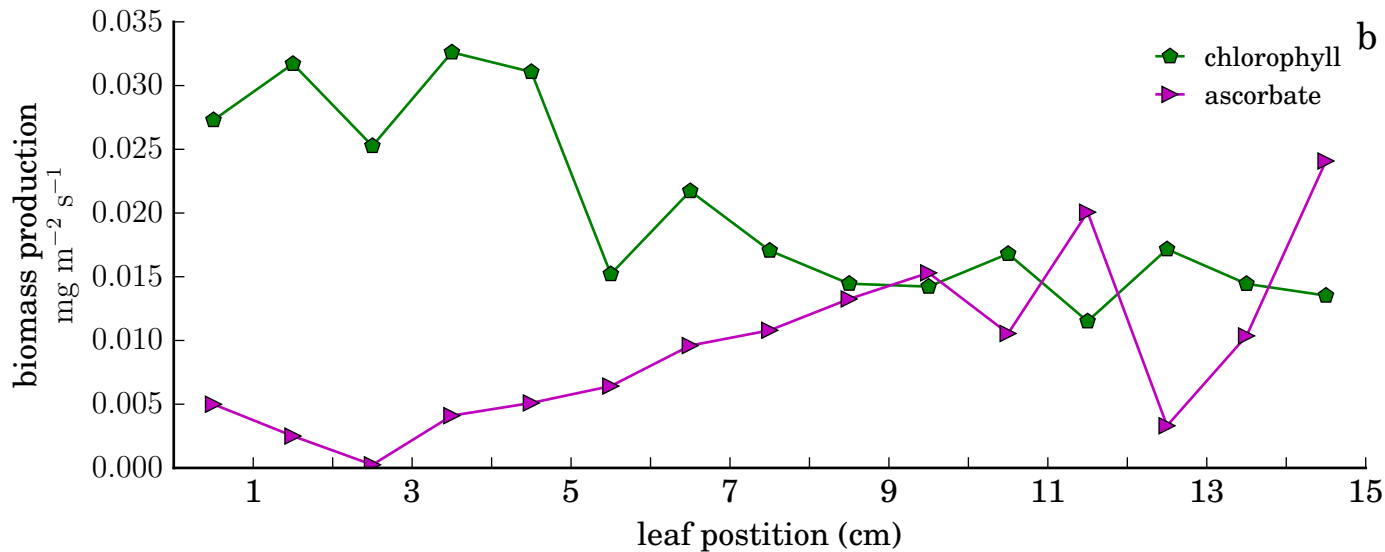

Supplement: S11 Fig — (a) Predicted production of cellulose, amino acids, nucleic acids, and lipids and fatty acids all show a pronounced peak at the base of the leaf and are higher in the predicted heterotrophic source region, consistent with the interpretation of this region as an area of active cell growth and division. (b) In contrast, predicted chlorophyll production is relatively steady along the leaf, while ascorbate production increases from the source-sink transition to the tip of the leaf. (PDF) [file pone.0151722.s011.pdf]
