# Supplementary material for: Multiscale Metabolic Modeling of C4 Plants: Connecting Nonlinear Genome-Scale Models to Leaf-Scale Metabolism in Developing Maize Leaves
Source: PLoS One. 2016 Mar 18;11(3):e0151722. doi: 10.1371/journal.pone.0151722 (PMC4807923; doi:10.1371/journal.pone.0151722)
Supplement: S1 Appendix — (PDF) [file pone.0151722.s012.pdf]

Generation of an FBA model from CornCyc

Eli Bogart   Christopher R. Myers

1 Outline

This file describes the of creation of a metabolic model for maize from CornCyc. It covers the creation of an SBML model with exchange and biomass reactions and limited subcellular compartmentalization which can successfully simulate the production of many biomass components and photosynthetic carbon dioxide assimilation, the adaptation of the biomass equation from iRS1563, some considerations in the process of expanding the model to describe interacting mesophyll and bundle sheath compartments, and some modifications made in response to preliminary fitting results.

Sections 2 through 8 explain in detail the process of constructing the underlying metabolic model at the one-cell level. Section 9 discusses in detail changes made to gene associations based on early data fitting results. Section 10 describes changes to the iRS1563 biomass equation. Section 11 discusses plasmodesmatal transport in the two-cell model. Filenames referred to are in the `model_development` subdirectory of the project source code (see S15 Protocol.)

2 Exporting the CornCyc FBA model from Pathway Tools

CornCyc 4.0 (Plant Metabolic Network (PMN), 2013) was obtained from the Plant Metabolic Network and upgraded from from Pathway Tools 16.5 to 17.0 locally.

The frame `PWY-561` was removed from the database because otherwise some of the reactions of that pathway were excluded from the FBA export, apparently due to a bug.

A simple FBA problem was solved using the Pathway Tools FBA functionality (Pathway Tools manual, 2013), producing an output file which includes all reactions in the FBA model Pathway Tools generates internally,

both those which are active in the solution to the FBA problem and those which are not. Note that this list of reactions is distinct from the list of reactions in the database itself; the Pathway Tools software prepares this set of reactions through an extensive process of excluding reactions which are unbalanced or otherwise undesirable while expanding reactions with classes of compounds as products or reactants into sets of possible specific instantiations which respect conservation of mass (Latendresse et al., 2012). Working with the Pathway Tools FBA reaction set (rather than, e.g, an SBML export of the CornCyc database) allows us take advantage of this pre-processing; however, it comes at the cost of needing to reintroduce into the FBA model many reactions which are present in the CornCyc database but are excluded from the FBA export for one reason or another.

Reaction data was extracted from the FBA output file, and reactions were translated to refer to species by their CornCyc frame ID (to allow easy reference to the database and comparison with previous work, and avoid possible ambiguities.) Reactions were then added and removed from the model as described below.

3 Discarding reactions

3.1 Polymerization reactions

Pathway Tools attempts to include an expanded representation of certain polymerization reactions in the exported FBA model, but this function is considered experimental (Pathway Tools manual, 2013); these reactions were ignored. Note that some reactions representing polymer growth were added manually later in the process.

3.2 ATPases

We removed all reactions from CornCyc which have the effective stoichiometry

```
{'ADP': 1.0, 'ATP': -1.0, 'PROTON': 1.0, 'WATER': -1.0,
'|Pi|': 1.0}
```

There are nine such reactions:

- `RXN-11109`,
- `3.6.4.6-RXN`,

- RXN-11135,
- RXN0-1061,
- ADENOSINETRIPHOSPHATASE-RXN,
- 3.6.4.4-RXN,
- 3.6.4.9-RXN,
- 3.6.4.5-RXN,
- 3.6.4.3-RXN

all treated as reversible by the Pathway Tools export procedure. Typically these are simplified representations of the metabolic effect of enzymes whose complete function is outside the scope of the database, as, for example, EC 3.6.4.3, the microtubule-severing ATPase.

In their place, we added a single generic ATPase reaction to represent cellular maintenance costs, etc., with no associated genes.

3.3 Reactions involving generic electron donors and acceptors

Numerous reactions in the database are written with generic representations of electron carrier species ('a reduced electron acceptor', 'an oxidized electron acceptor'). Most of these reactions are outside the areas of emphasis of the model (e.g., brassinosteroid biosynthesis), have no curated pathway assignment, or also appear in forms which do specify the electron carrier species (e.g., the generic nitrate reductase reaction, NITRATEREDUCT-RXN, vs NITRATE-REDUCTASE-NADH-RXN,) and so could be safely neglected. A small set of exceptions identified in early drafts included reactions of fatty acid synthesis, handled as discussed below, and proline dehydrogenase, RXN-821, catalyzed by a mitochondrial-membrane-bound flavoprotein which donates electrons directly to the mitochondrial electron transport chain (Elthon and Stewart, 1982). Because we have not thoroughly compartmentalized amino acid metabolism, we implemented this reaction as donating electrons to NAD^+ instead.

3.4 Duplicates

A number of other reactions were removed because they appeared to be exact (possibly unintentional) duplicates, down to gene associations, of other

reactions in the database; or because they were being replaced by modified forms as discussed below. These are given in `reactions_to_remove.txt`.

3.5 Non-metabolic reactions

A number of reactions present in CornCyc were removed because the database indicated, e.g. through the Enzyme Commision summary for the relevant EC number, that they were primarily involved in extrametabolic functions (e.g., cell movement, regulation). These included the GTPases `RXN-5462`, `3.6.5.2-RXN`, and `3.6.5.5-RXN`.

3.6 Glucose-6-phospate

In the reduced model (discussed below) only one reaction, myo-inositol-1-phosphate synthase, consumes the generic glucose-6-phosphate species, rather than alpha-G6P or beta-G6P. To ensure that this reaction was appropriately connected to other G6P producing and consuming reactions we manually split it into two instances, one for alpha-G6P and one for beta-G6P.

3.7 UDP-glucose

For apparently all reactions in CornCyc involving UDP-glucose, the instantiation procedure produced one version involving generic UDP-D-glucose and one version involving UDP-alpha-D-glucose, the only child of the UDP-D-glucose class. UDP-alpha-D-glucose participated in almost no reactions other than these instantiations (in the reduced model, described below, only one: UDP-sulfoquinovose synthase, EC 3.13.1.1). As such there is little to distinguish the generic and specific versions of the reactions, which add complexity to the model and degeneracy to optimization predictions without providing significant information about the function of the system, so we removed the specific versions and changed the UDP-sulfoquinovose synthase to act on a generic UDP-D-glucose substrate.

4 Minor revisions to achieve basic functionality

4.1 Mitochondrial electron transport chain

The CornCyc representation of the mitochondrial electron transport pathway (`PWY-3781`, plus the mitochondrial ATPase (`ATPSYN-RXN`, EC 3.6.3.14))

was adjusted. Some reactions excluded from the initial Pathway Tools export because the balance state of reactions involving cytochrome C could not be determined were readded manually; ubiquinones/ubiquinols were uniformly represented as ubiquinone-8/ubiquinol-8, and compartments were assigned to reactants and products to properly represent the transport of protons between the mitochondrial matrix and the mitochondrial intermembrane space. In CornCyc, as in MetaCyc and other related databases, transport of protons across the membrane is represented explicitly for complex I but not for complex III and complex IV; in agreement with the standard description of mitochondrial electron transport (see, e.g., Brownleader et al., 1997) proton transport was added to these reactions with a stoichiometry of 2 H+/e- for complex III and 1 H+/e- for complex IV. The stoichiometry of complex IV was further adjusted to include the H+ from the mitochondrial matrix that binds to oxygen to form water.

4.2 Photosynthesis: light reactions

Similarly, some modifications were made to the light reactions of photosynthesis (`PWY-101`). Reactions involving plastocyanins were not exported and were added manually; a chloroplastic ATP synthase and a reaction describing cyclic electron transport around PS I were added; and the stoichiometry of proton transport was adjusted in accordance with recent literature, assuming a Q cycle and ratio of 14 H+/3 ATP for the chloroplast ATP synthase (Allen, 2003).

Reduction of oxygen to superoxide at photosystem I (the Mehler reaction) was added to allow flux through the pathways of chloroplastic reactive oxygen species detoxification: superoxide dismutase and the ascorbate-glutathione cycle, including a reaction representing the direct, non-enzymatic reduction of monodehydroascorbate by ferredoxin (Asada, 1999; Foyer and Harbinson, 1997).

4.3 Key reactions in biomass component production and nutrient uptake

Several components of biomass required either manual adjustment of reactions from the database or the addition of abstract synthesis reactions summarizing the behavior of pathways which could not easily be represented in more detail.

4.3.1 Starch

Starch synthase (GLYCOGENSYN-RXN) is not exported from CornCyc by default (it is a polymerization reaction, and marked as unbalanced in the PGDB); it was added manually in a form that produces the equivalent of one 1,4-alpha-D-glucan subunit.

The starch branching enzyme EC 2.4.1.18 (RXN-7710) is not exported from CornCyc by default (one reactant, starch, has an unspecified structure); it was added manually as

$$\text{a 1,4-alpha-D-glucan subunit} \rightarrow \text{an amylopectin subunit}$$

Note that this stoichiometry is not intended to suggest that the branching enzyme introduces branches at each subunit.

CornCyc provides a detailed reconstruction of the reactions of starch degradation (PWY-6724) which is by nature difficult to convert to a form suitable for FBA calculations, as many of the stoichiometry coefficients are undefined. To incorporate the effects of the glucan-water and phosophoglucan-water dikinases, for example, we would need to specify how many glucosyl residues must be phosphorylated (and then dephosphorylated) to produce "an exposed unphosphorylated, unbranched malto-oligosaccharide tail on amylopectin" of a given length; modeling the release of maltose from that tail would require an estimate of the typical unbranched length of such tails, etc. Rather than estimate average values for these parameters, we divide the reactions of the pathway into two types: those which condition starch for depolymerization , and actual depolymerization reactions. The first class (the dikinases above plus isoamylase) share the abstract stoichiometry

$$\text{a starch subunit} \rightarrow \text{an exposed starch subunit}$$

(neglecting any ATP costs), while the second class (beta amylase and disporportionating enzyme) convert exposed starch subunits to sugars appropriately.

The beta-maltose releasing reactions of the starch degradation pathway in CornCyc have no associated genes. We temporarily associated these reactions with the beta amylase record in the database (RXN-1827, EC 3.2.1.2) pending further review.

During transient starch degradation, beta-maltose and glucose are exported into the cytosol, where maltose is split, releasing one glucose molecule and donating one glucosyl residue to a cytosolic heteroglycan, from which it may be released in turn as glucose-1-phosphate (Fettke et al., 2009). In

Arabidopsis, specific enzymes (DPE2 and PHS2) are known to be implicated in this process (Streb and Zeeman, 2012). In simulations with this CornCyc-based FBA model we find the typical mode of breakdown of cytosolic maltose is to alpha-D-glucose and alpha-D-glucose-1-phosphate via `AMYLOMALT-RXN`,

$$\text{maltotriose} + \text{beta-maltose} \rightarrow \text{maltotetraose} + \text{beta-D-glucose}$$

and `RXN0-5182`,

$$\text{maltotetraose} + \text{phosphate} \rightarrow \text{maltotriose} + \text{alpha-D-glucose-1-phosphate}$$

effectively the standard pathway but with maltotriose/maltotetraose playing the role of the cytosolic heteroglycan pool. This approximation leads to a reasonable effective stoichiometry but it is possible that the genes associated with these reactions do not accurately represent the genes involved in the true underlying process; we have not systematically looked for maize counterparts of the Arabidopsis genes, for example.

4.3.2 Cellulose

The UDP-forming cellulose synthase, EC 2.4.1.12, is not exported from CornCyc by default (it is a polymerization reaction, and marked as unbalanced in the PGDB); it was added manually in a form that produces the equivalent of one subunit.

4.3.3 Hemicellulose

Similarly, the following hemicellulose polymerization reactions were added manually:

- 1,4-beta-D-xylan synthase, EC 2.4.2.24,
- reactions `RXN-9093` (EC 2.4.2.-) and `RXN-9094` (EC 2.4.1-), representing the addition of arabinose and glucuronate to xylan to form arabinoxylan and glucuronoxylan respectively (note that the corresponding subunits notionally consist of one xylan subunit plus arabionose/glucuronate),
- glucomannan synthase, EC 2.4.1.32,
- `RXN-9461` (EC 2.4.2.39), representing the addition of xylose to a glucan (as implemented, cellulose) to form xyloglucan (again, the corresponding effective subunit corresponds to one glucan subunit plus xylose)–

note this representation ignores the previous step in CornCyc's xyloglucan biosynthesis pathway, xyloglycan 4-glucosyltransferase (EC 2.4.1.168).

In addition to these explicit descriptions of hemicellulose formation from CornCyc, we added generic reactions representing the donation of the following sugar residues from activated donor molecules to unspecified generic polysaccharides:

- arabinose (from UDP-L-arabinose)
- galactose (from GDP-L-galactose)
- galacturonate (from UDP-D-galacturonate)
- glucose (from UDP-glucose)
- glucuronate (from UDP-D-glucuronate)
- mannose (from GDP-alpha-D-mannose)
- xylose (from UDP-alpha-D-xylose)

These reactions allow the model to represent flux of these sugars towards hemicelluloses or other polysaccharides without explicit synthesis pathways in CornCyc, or the construction of a hemicellulose term in the biomass equation in terms of the overall composition of hemicellulose without reference to specific synthesis reactions, as in our adaptation of the biomass reaction of Saha et al. (2011) (see the biomass reaction discussion, below.)

4.3.4 Miscellaneous cell wall components

The following additional cell wall comoponent production reactions from CornCyc were added manually:

- `2.4.1.43-RXN`, representing the formation of homogalacturonan from galacturonate
- `RXN-9589` (EC 2.4.2.41), representing the addition of xylose to homogalacturonan to form xylogalacturonan (note the resulting xylogalacturonan subunit notionally consists of one galacturonate plus xylose)
- `13-BETA-GLUCAN-SYNTHASE-RXN` (EC 2.4.1.12), representing the formation of callose from glucose.

Suberin production is not represented in CornCyc in detail but pathways for the synthesis of three key precursors, N-feruloyltyramine, octadecenedioate, and docosanediotate, are provided. Sinks for N-feruloyltyramine and octadecenedioate were added to the model to represent the flow of material towards suberin production; docosanedioate was neglected because no genes are associated with the reactions of its synthesis pathway. N-feruloyltyramine may be produced from trans-caffeate via either ferulate or caffeoyl-CoA; the branch through ferulate was initially dropped from the reduced version of the model used for data analysis because it relies on trans-feruloyl-CoA synthase, EC 6.2.1.34, which has no associated genes, but it was preserved in subsequent versions of the model because high expression levels for caffeate O-methyltransferase suggest this branch is indeed active.

(In CornCyc, the tyramine N-feruloyltransferase that produces N-feruloyl-tyramine from feruloyl-CoA could also catalyze the production of other hydroxycinnamic acid tyramine amides (cinnamoyltyramide, sinapoyltyramide, p-coumaroyl-tyramine) but we have neglected these for now.)

4.3.5 Fatty acids and lipids

Plant fatty acid and lipid biosynthesis is rich in complexity (see, e.g., Li-Beisson et al., 2013), and attempting to describe it in the FBA model at the level of detail at which it is currently understood would require a daunting number of reactions among the species representing the combinations of lipid head groups and acyl chains. Though CornCyc presents some pathways of lipid metabolism at such a high resolution, we have adopted a simplified approach which aims to include enough detail to allow the model to:

- predict based on RNA-seq data the total flow of biomass into fatty acids and lipids
- coarsely predict differences in the types of lipids and fatty acids produced, based on RNA-seq data
- approximately preserve the iRS1563 biomass equation.

The model describes in detail the sequence of reactions by which fatty acids up to lengths of 16 and 18 are synthesized in the chloroplast (though currently these reactions occur in the cytoplasmic compartment!), and the formation of oleate (as oleoyl-ACP) by the stearoyl-ACP desaturase (`PWY-5156`; Ohlrogge and Browse (1995); Li-Beisson et al. (2013)). In practice, these fatty acids may then enter the 'prokaryotic' pathway of glycerolipid synthesis in the chloroplast or leave the chloroplast and enter the 'eukaryotic'

pathway of glycerolipid synthesis in the endoplasmic reticulum, with further desaturation of the acyl chains occurring after their incorporation into lipids.

We simplify this process by effectively decoupling the synthesis of different types of lipids (as distinguished by head groups) from the desaturation of their associated acyl chains. Reactions from lipid synthesis pathways are implemented as if all lipid species had one 16:0 and one 18:1 acyl chain, by implementing the glycerol-3-phosphate O-acyltransferase and 1-acylglycerol-3-phosphate O-acyltransferase reactions (RXN-10462 and 1-ACYLGLYCEROL-3-P-ACYLTRANSFER-RXN), written in the database with generic acyl-acp substrates, with oleoyl-ACP and palmitoyl-ACP as substrates respectively. (This corresponds to the prokaryotic pathway; in the eukaryotic pathway oleoyl-CoA and palmitoyl-CoA would supply the acyl groups for diacylglycerol formation instead (Li-Beisson et al., 2013) However the same genes are associated with the reactions of diacylglycerol synthesis in the two pathways (`PWY-5667`; `PWY0-1319`) in CornCyc and so they cannot be distinguished based on expression data alone; we have chosen one arbitrarily.)

This supply of diacylglycerol is sufficient to allow, without further modification to the CornCyc FBA export, the synthesis of a variety of lipids, including:

- phosphatidylcholine, phosphatidylethanolamine, phosphatidylglycerol, phosphatidylinositol;
- sulfoquinovosyldiacylglycerol.

UDP-glucose epimerase is exported from CornCyc in the UDP-glucose-producing direction by default; we allowed it to run in the reverse direction as well, consistent with literature evidence (Dörmann and Benning, 1998; BRENDA, 2013), which allowed the production of mono- and digalactosyl-diacylglycerol.

In sphingolipid metabolism, dihydrosphingosine, 4-hydroxysphinganine and sphinganine 1-phosphate may be produced, and sink reactions were added for them. Production of the ceramides and their derivatives would require the choice of a particular fatty acid source for the sphinganine acyltransferase, written by default with the generic substrate 'a long-chain acyl-coA'; per the CornCyc description page for `PWY-5129`, in leaf sphingolipids C20 to C26 fatty acids are typical. Currently, the FBA model lacks a detailed implementation of production of very long chain fatty acids by elongation (a

generic representation is present in CornCyc), so no supply of C20-26 fatty acids is available. We have deferred this issue to future work.

Separately, we model the desaturation of oleate to linoleate and linolenate and palmitate to palmitoleate. These (along with palmitate and stearate) are the fatty acid components of the iRS1563 biomass reaction, which originally incorporated them as triglycerides; our modified biomass equation consumes free fatty acids, rather than attempt to specify the precise ratios in which they are to be found in different lipid species in the leaf.

The CornCyc pathways for linoleate and linolenate produce them as lipid linoleoyl groups and lipid linolenoyl groups respectively, incorporated in generic lipid molecules; to allow these reactions to balance, and to provide linoleate and linolenate for the biomass reaction, we added lipases which release free linoleate/linolenate from the lipid linoleoyl and lipid linolenoyl groups, regenerating the pool of generic 'lipid' species (which participate only in the linoleate pathway, within the FBA model.) Note, however, that other reactions within the model but outside the indicated synthesis pathways are capable of producing linoleate and linolenate as well.

CornCyc includes no complete pathway for the production of palmitoleic acid; as there is experimental evidence it is produced in maize leaves (see the discussion of the biomass equation, below) we introduced the acyl-ACP Δ9-desaturase reaction from the palmitoleate biosynthesis pathway of AraCyc (`RXN-8389`, 1.14.99.-), producing palmitoleoyl-ACP from palmitoyl-ACP (Plant Metabolic Network (PMN), 2014a), which restores this functionality (in combination with the palmitoleoyl-ACP hydrolase, `RXN-9550`, which is present in CornCyc.) Note that there is some evidence that the stearoyl-ACP desaturase enzyme may also catalyze this reaction (Gibson, 1993).

The oleoyl-acyl carrier protein hydrolase (EC 3.1.2.14) from CornCyc is unbalanced with respect to hydrogen; a version with an additional proton on the right hand side was added manually.

The Δ9-desaturase and the desaturases producing linoleate and linolenate (`RXN-9667` and `RXN-9669`) were written originally with generic electron donor and acceptor species. Initial review of the extensive literature on plant fatty acid desaturation suggests that the electron source for desaturases depends on their location within the cell, with chloroplastic desaturases accepting electrons from ferredoxin while desaturases in the endoplasmic reticulum accept electrons from NADH via cytochrome b5 or fused cytochrome domains see, eg, (see, e.g., Sperling et al., 1995; Harwood, 1996; Shanklin and Cahoon, 1998). As discriminating between chloroplastic and extrachloroplastic fatty acid desaturation is not a high priority for the model, NADH

was used as the sole electron donor for all three of these reactions.

The ferredoxin-dependent stearoyl-ACP desaturase `RXN-7903`, not exported from the database by default because it is marked as unbalanced, was added in a form adjusted for hydrogen and charge balance. Ferredoxin-NADP oxidoreductase was made reversible to ensure NADPH can drive this reaction in the dark, as is observed (Shanklin and Cahoon, 1998).

4.3.6 Nucleic acids polymerization

Reactions representing the pyrophosphate-releasing incorporation of (d)NTPs into RNA and DNA were added and associated with the DNA-directed DNA polymerase and DNA-directed RNA polymerase reactions in the database. (In each case, it is assumed that all nucleotides occur with equal frequency.)

4.4 Ascorbate-glutathione cycle

To allow the NADPH-monodehydroascorbate reductase reaction to function in the cycle as curated, we split the L-ascorbate peroxidase reaction (EC 1.11.1.11) into its two subreactions, which by default are not exported in the FBA problem.

4.5 Gamma-glutamyl cycle

The gamma-glutamyltransferase was lumped together with `GAMMA-GLUTAMYL-CYCLOTRANSFERASE-RXN`, originally written in terms of the instanceless class '`L-2-AMINO-ACID`' which appeared in no other stoichiometries in the FBA export, and the dipeptidase `RXN-6622`, which is the only reaction that can consume the cysteinylglycine product of the gamma-glutamyltransferase, forming a combined reaction which can carry flux. The combined reaction retained the gene associations of the gamma-glutamyltransferase, as the other two reactions have no associated genes.

4.6 Methionine synthesis from homocysteine

The methionine synthase reaction of CornCyc's methionine biosynthesis pathway, `HOMOCYSMET-RXN`, EC 2.1.1.14, specifically requires 5-methyltetrahydropteryltri-L-glutamate as a cofactor. Polyglutamylation of folates is present in CornCyc in an abstract representation (with tetrahydrofolate synthase catalyzing the addition of a glutamyl group to a 5-methyltetrahydropteryl with n glutamyl groups); we have not converted this into an explicit representation in the FBA model. Instead, `HOMOCYSMETB12-RXN`,

EC 2.1.1.13, acts to produce methionine from homocysteine; the effects of this possible inaccuracy on the behavior of the rest of the network should be limited.

4.7 Basic import and export

The following species are given overall import/export reactions:

- `WATER`
- `CARBON-DIOXIDE`
- `OXYGEN-MOLECULE`
- `PROTON`
- `NITRATE`
- `SULFATE`
- `|Pi|`
- `|Light|`
- `MG+2`

These reactions exchange species inside the cell with species in meaningfully labeled compartments where possible (eg, oxygen and CO_2 are exchanged with the intercellular air space, mineral nutrients with the xylem, etc.)

In addition, to facilitate exchange among compartments in the whole-leaf model, a number of exchanges with a phloem compartment were set up: these included sucrose, glycine (as a representative of the amino acids detected in maize phloem sap by Ohshima et al (Ohshima et al., 1990),) and the potential phloem sulfur transport compound glutathione (Bourgis et al., 1999).

Note that these reactions should be inactive, or restricted to the exporting direction only, when not modeling transport within the leaf (except for sucrose, where a free supply should be allowed in heterotrophic conditions.)

4.8 Defining the biomass components

Two types of biomass reactions are added to the model:

- Sinks for individual species, for simulations (e.g, fits to RNAseq data) where the relative rates of production of different components are unknown. These reactions are listed in S22 Appendix; additionally, the species given such sinks are listed in `biomass_components.txt` in the `model_development` subdirectory of the project source code (see S15 Protocol.)

- A set of reactions producing a combined biomass species, made up of assorted components in fixed proportions, for simulations where the maximum rate of production of biomass is of interest, and an approximately realistic biomass composition needs to be enforced directly. These reactions were taken with minor modifications from Saha et al. (2011). They are listed in S22 Appendix, additionally, their adaptation is described below (section 10) and they are listed in `adapted_irs1563_biomass.txt` in the `model_development` subdirectory of the project source code (see S15 Protocol.)

To conceptually and practically separate these types of biomass reactions, which in general should not both be active in any one calculation, the biomass species they produce are located within two separate abstract biomass compartments in the SBML model.

In general, the biomass sink reactions have no gene associations, but an exception was made for the twenty reactions representing incorporation of amino acids into protein, which inherit the gene associations of the corresponding tRNA ligase reactions in CornCyc. (In principle these could be distinguished from sink reactions representing the expansion of free amino acid pools as cells grow and divide, but we have ignored this issue for now.)

Note that, to support the adapted iRS1563 biomass equation, a reaction representing the production of free galactose from GDP-L-galactose was introduced (otherwise, release of galactose from UDP-galactose was catalyzed by two reactions in the pathways of indole-3-acetyl-ester conjugate biosynthesis and indole-3-acetate activation, likely not a major route for carbohydrate production.) Free galactose is not included in the individual biomass species used for data fitting.

5 Compartmentalization

Approaches differ to the subcellular compartmentalization in FBA models of eukaryotes, ranging from the assignment of compartments to a few key pathways known to function primarily outside the cytosol, as in the mitochondrial and chloroplastic "modules" of AraMeta (Poolman et al., 2009) and RiceMeta (Poolman et al., 2013) to the extremely comprehensive, data-driven approach of Mintz-Oron et al. (2012). Here, we did not attempt to comprehensively assign reactions to their proper compartments; instead, we started with a modular approach similar to Poolman et al. (2013) in which some core metabolic pathways were compartmentalized (in our case, the TCA cycle and mitochondrial electron transport chain in the mitochondrion, the light reactions of photosynthesis, Calvin cycle, and some reactions of the C4 and photorespiratory pathways in the chlorophyll, and some reactions of the photorespiratory pathway in the peroxisome, with transport reactions added as necessary.)

We then refined the compartment assignments of other reactions and pathways as needed to permit key metabolic functions and compartmentalize a limited number of additional reactions whose incorrect assignment to the cytosol we judged particularly likely to lead to misleading results.

More details on individual compartmentalization choices and transport reactions are given below.

5.1 Intracellular transport

Sources (beyond those detailed below) informing the addition of intracellular transport reactions in the model included the transport reactions present in AraMeta (Poolman et al., 2009), reviews of photorespiratory metabolism with attention to compartmentalization (Reumann and Weber, 2006; Foyer et al., 2009), a review of chloroplast transporters by Weber and Linka (2011), and a review of transport processes in C4 photosynthesis by Bräutigam and Weber (2011).

In most cases we have not tried to reflect the mechanisms of the transport systems, where those are known, in any detail (exceptions include the triose phosphate-phosphate and PEP-phosphate transporters across the chloroplast envelope), nor have we associated genes with the transporters, even when they are known. Future work should pay greater attention to this aspect of the system.

5.2 Photorespiratory pathway

Following Douce and Heldt (2000) we assumed that reducing power was supplied to the peroxisome through an oxaloacetate-malate shuttle and NAD(H)-dependent malate dehydrogenase, and added an oxaloacetate-malate antiporter and a copy of `MALATE-DEH-RXN` to the peroxisome. Reactions of the pathway were localized following Reumann and Weber (2006) and Foyer et al. (2009). Note that glycine decarboxylase was assigned exclusively to the mitochondrion, while serine hydroxymethyltransferase was present in both the mitochondrion and the cytoplasm, where it plays a role in one-carbon metabolism (Hanson and Roje, 2001).

5.3 Various ferredoxin-consuming pathways

The model includes several pathways or reactions (e.g., sulfite and nitrite reduction and the chlorophyll cycle) which rely on ferredoxins for reducing power, and are localized to the chloroplast, where, in the light, reduced ferredoxins may be supplied by the photosynthetic electron transport chain.

Rather than assign the reactions of these pathways to compartments appropriately, we added a reaction exchanging reduced ferredoxins and oxidized ferredoxins across the chloroplast boundary to supply ferredoxin-driven pathways in the cytosol. We emphasize that this is a convenient simplification and is not intended to represent a realistic mechanism.

5.4 Ascorbate production

The L-galactonolactone dehydrogenase responsible for the final step of the ascorbate production pathway in CornCyc reduces cytochrome C and has been experimentally localized to the mitochondrial inner membrane, with its catalytic site facing outwards, into the intermembrane space (Bartoli et al., 2000). As the outer membrane is generally permeable to small molecules we have treated this reaction as acting directly on cytoplasmic galactonolactone and ascorbate. A sink for ascorbate as a biomass component was added, as it is found in substantial quantities in leaves (see, e.g., Foyer et al., 1983; Smirnoff, 1996).

5.5 Ascorbate-glutathione cycle

This cycle is present in multiple cellular compartments; in the model we included only cytosolic and chloroplastic instances (of which only the chloroplastic was ultimately expected to be relevant, as there was no supply of

superoxides in the cytosol.) Note that none of the genes associated with monodehydroascorbate reductase could be assigned to the chloroplast under the rules described below: two had curated location in the peroxisome while GRMZM2G320307 had no curated location and TargetP prediction of mitochondrial (GRMZM2G320307_P01) and cytoplasmic (GRMZM2G320307_P02, GRMZM2G320307_P03) locations. Reduction of monodehydroascorbate may also proceed non-enzymatically (see above) so this (enzymatic) reaction was removed from the chloroplast in favor of direct reduction by ferredoxin.

6 Gene associations for compartmentalized reactions

Where a reaction was present in more than one compartment– that is, when two or more reactions in different compartments were associated with the same reaction record in CornCyc– we examined the genes associated with those reactions in CornCyc and assigned them to the instance of the reaction in the most appropriate compartment, as far as possible.

Where the Plant Proteome Database (Sun et al., 2009) provided manually curated location assignments for genes, those were used; otherwise, we used automatic location predictions by TargetP (Emanuelsson et al., 2000) or in some cases referred to the gene's annotation (both also provided by PPDB.) In general we assumed the appropriate location for a gene product was the cytoplasmic compartment absent a specific prediction of localization in the chloroplast, mitochondrion, or peroxisome. Where proteins were predicted to occur in a compartment where an no instance of a particular reaction was present, those gene associations were generally dropped from the model.

When a gene was associated with a reaction in more than one compartment and also a reaction present in only one compartment, in general the association with the reaction in only one compartment was dropped, except for reactions which we believed based on literature evidence (including comments in CornCyc and PPDB) were assigned to the cytoplasmic compartment only because our compartmentalization process was incomplete.

Some details on the judgment calls made in this process are provided in the comments to the file gra_overrides.txt; we comment here on a few unusual cases.

6.1 NADH dehydrogenases

Cyclic electron transport around Photosystem I may occur through the chloroplast NADH dehydrogenase complex or an alternate pathway which in Arabidopsis involves PGR5 (Munekage et al., 2004; Shikanai, 2007). In C3 plants the PGR5-dependent pathway may play the major role in tuning the photosynthetic ATP/NADPH ratio, while the NADH dehydrogenase pathway is implicated in stress responses (Shikanai, 2007). In contrast, in C4 plants the expression of the chloroplast NADH-dehydrogenase appears to correlate with photosynthetic ATP demand, while PGR5 expression does not, suggesting it is the NADH-dehydrogenase CET pathway which allows increased the increased ATP production required by the C4 system (Takabayashi et al., 2005). Thus, genes associated in CornCyc with the NADH dehydrogenase reaction for which a chloroplast location was predicted were reassociated with the model's cyclic electron transport reaction (despite the fact that our somewhat abstract cyclic electron transport reaction may not accurately represent the biochemistry of the NADH-dependent pathway.)

6.2 Pyruvate dehydrogenases

In practice, pyruvate dehydrogenase complexes are found in the mitochondrion and chloroplast, but here we have not fully compartmentalized the chloroplastic pyruvate dehydrogenase and the pathways it supplies, instead leaving it in the cytosol. Thus, genes associated with the reactions of the complex with predicted chloroplast localization were associated instead with the cytosolic version. Genes with no curated or predicted location were left associated with both forms (splitting their expression data between them, in the fitting process.)

7 Testing and consistency checking

The compartmentalized single-cell model was checked in detail for conservation violations by testing the feasibility of net production or consumption of a unit of each internal species with all external transport and biomass sink reactions suppressed. Where such production was found feasible, the reactions involved were carefully inspected and stoichiometry coefficients adjusted to restore balance if necessary. In practice, this led only to the correction of erroneous reactions added by hand; as expected, no balance issues were found with reactions exported from CornCyc.

In the final version, no such unrealistic processes are possible in the

model under normal conditions. (Note that the species representing light input may be consumed in isolation, but the use of light energy to drive a futile cycle is not unrealistic, though we have not examined the details of the process found by the consistency checker in any detail.) Of course, demonstrating that no such production/consumption is feasible does not guarantee that all reactions in the model are properly balanced.

Testing also verified that all individual biomass sink reactions, and the combined biomass reaction, could proceed at nonzero rates.

8 SBML export

8.1 Component names

SBML distinguishes a component's name from its ID. Reactions and species in the SBML model were given name attributes according to the by calling the Pathway Tools `get_name_string` function on the frames in the database from which they derive, if any. The IDs of the SBML components were derived from the frame handles, replacing special characters with underscores as necessary to conform to the SBML sID standard.

Note that for some reactions in CornCyc, the result of `get_name_string` is an EC number different from the EC number indicated by the label of the frame (e.g, `2.7.1.133-RXN`, for which 'EC 2.7.1.159' is returned.) The frame in CornCyc (if any) from which each reaction in the SBML model is ultimately derived is preserved as a comment in the reaction's Notes element, to resolve any ambiguity.

8.2 Gene annotations

Each reaction in the FBA model associated with a particular parent frame in CornCyc was given an association rule that combined all genes associated with that reaction in CornCyc, as well as all genes associated with all generic reactions of which the parent reaction is a specific form, in a logical 'or' relationship, stored in the reaction's Notes element per the COBRA standard.

9 Model refinement

9.1 Phosphoribulokinase

In early attempts to fit the model to the leaf gradient data, high costs were associated with the mesophyll phosphoribulokinase reaction in the source tissue when the bundle sheath CO_2 level was high. We noted that in CornCyc 4.0 several genes were associated with both PRK and glyceraldehyde-3-phosphate dehydrogenase. To clarify the role of these genes we referred to annotations in the Plant Proteome Database (Sun et al., 2009) and best hits in the Conserved Domain Database (Marchler-Bauer and Bryant (2004), accessed through NCBI.) Of the eight genes associated with PRK in CornCyc, three (`GRMZM2G039723`, `GRMZM2G337113`, `GRMZM2G162845`) appeared to encode GAPDH enzymes (per PPDB annotations and the presence of `Gp_dh_N` and `Gp_dh_C` domains), three (`GRMZM2G162529, GRMZM2G463280, GRMZM2G026024`) appeared encode to encode genuine phosphoribulokinases (per PPDB annotations and the presence of PRK domains), and two appeared to encode CP12-type regulatory proteins, with no obvious evidence for any individual protein sharing more than one of these roles. The regulatory role of CP12 does involve forming a complex with PRK and GAPDH, but this reduces, rather than enhancing or enabling, their individual activities (Lopez-Calcagno et al., 2014). We removed the PRK associations of the GAPDH and CP12 genes from our model. PPDB assigned these three GAPDH genes to a plastidic location based on experimental evidence, so we associated them with those reactions exclusively (removing associations with the cytosolic instances of EC 1.2.1.13 and/or EC 1.2.1.12.)

10 Biomass equation

We developed a biomass equation following that used by Saha et al. (2011). Our calculations are based on supplementary file S4 of that paper[1], in particular sheet 2, 'Biomass_rxn'.

That sheet derives a biomass equation corresponding to the production of one gram of plant dry weight, based on literature data on biomass composition; the description is divided into subreactions forming (e.g.) 'nitrogenous compounds', 'lignin', etc., which then participate in an overall biomass reaction.) The units of the stoichiometric coefficients are mmol.

[1] specifically, `journal.pone.0021784.s004.xls`, as downloaded from the PLoS One web site 20 November 2013

We have adopted most of the biomass composition assumptions of Saha et al wholesale, with gratitude for their efforts in compiling this data from the literature. However, we have made some minor adjustments, resulting in a different overall stoichiometry for biomass production.

10.1 Fatty acids

Saha et al represent the total lipid/fatty acid contribution to biomass as a pool of triglycerides in proportions apparently based on a maize oil measurement and thus probably reflective of seed triglyceride composition.

We substitute measurements of the fatty acid content of mature maize leaf membrane lipids (Rizov and Doulis, 2000) and write a biomass subreaction which consumes the relevant free fatty acids (rather than their derivatives in the form of triacylglycerols, membrane lipids, etc.,) as shown in Table 10.1.

Fatty acid	CornCyc compound	mol. wt. (g/mol)	mole fraction
palmitic	`PALMITATE`	255.42	0.104
palmitoleic	`CPD-9245`	253.4	0.056
stearic	`STEARIC_ACID`	283.47	0.011
oleic	`OLEATE_CPD`	281.46	0.044
linoleic	`LINOLEIC_ACID`	279.44	0.132
linolenic	`LINOLENIC_ACID`	277.43	0.646

Table 1: Fatty acid proportions in biomass.

Weighting the molecular weights by the mole fractions, we find one mole of fatty acid in appropriate proportions weighs 272.4 g. Dividing the mole fractions by the overall molar weight and multiplying coefficients by 1000 to convert to millimoles, we arrive at the final equation:

0.382 `PALMITATE` + 0.206 `CPD-9245` + 0.04 `STEARIC_ACID` +
0.162 `OLEATE_CPD` + 0.485 `LINOLEIC_ACID` + 2.372 `LINOLENIC_ACID`
= `fatty_acids_biomass`

where the left-hand side represents 1 g.

Fractions add to less than 1.0 because we ignore trace (mole fraction $\leq$ 0.01) amounts of C14:0 and C20:0 fatty acids. Note that the leaf fatty acid composition is known to change along the developmental gradient, so specifying any single composition is an approximation; see Leech et al. (1973).

10.2 Hemicellulose

We adopted the hemicellulose production reaction as is, using the species added to the model for this purpose, 'polysaccharide_[sugar]_unit'. The resulting equation is:

0.548 polysaccharide_arabinose_unit +
1.248 polysaccharide_xylose_unit +
0.301 polysaccharide_mannose_unit +
0.144 polysaccharide_galactose_unit +
3.254 polysaccharide_glucose_unit +
0.166 polysaccharide_galacturonate_unit +
0.166 polysaccharide_glucuronate_unit = hemicellulose_biomass.

10.3 Total carbohydrates

We recalculated the stoichiometries of the carbohydrate-producing reaction to account for the differing molecular weight of our representation of cellulose ('CELLULOSE_monomer_equivalent', effectively a glucose molecule), account for the fact that one unit of hemicellulose represents one gram, not one (milli)mole, and express pectin in terms of polysaccharide_galacturonate_unit, reflecting a belief that UDP is released in the formation of pectin from UDP-D-galacturonate, rather than retained in the polymer (Plant Metabolic Network (PMN), 2014d).

It is not clear what form the 'mannose' referred to by Penning de Vries et al should be assumed to take, as free mannose is not found in plants under most circumstances (see, e.g., Herold and Lewis, 1977; Schnarrenberger, 1990; Plant Metabolic Network (PMN), 2014b) Here we somewhat arbitrarily choose mannose-6-phopshate.

Component	Species in model	unit wt (mg)	wt fraction	units/g product
Ribose	RIBOSE	150.053	0.010	0.067
Glucose	GLC	180.063	0.050	0.278
Fructose	FRU	180.063	0.020	0.111
Mannose	MANNOSE-6P	258.120	0.010	0.039
Galactose	GALACTOSE	180.063	0.010	0.056
Sucrose	SUCROS	342.116	0.050	0.146
Cellulose	CELLULOSE_monomer_equivalent	180.160	0.400	2.220
Hemicellulose	hemicellulose_biomass	1000.000	0.400	0.400
Pectin	polysaccharide_galacturonate_unit	193.130	0.050	0.259

Table 2: Carbohydrate species in biomass.

Table 10.3 shows the calculation, resulting in the equation:

0.067 RIBOSE + 0.278 GLC + 0.111 FRU + 0.039 MANNOSE-6P +
0.056 GALACTOSE + 0.146 SUCROSE + 2.220 CELLULOSE_monomer_equivalent

+ 0.400 hemicellulose_biomass + 0.259 polysaccharide_galacturonate_unit = carbohydrates_biomass.

10.4 Organic acids

We adopt this reaction as is. In the terminology of our model, the resulting equation is:

0.556 OXALATE + 0.676 GLYOX + 1.515 OXALACETIC_ACID + 0.746 MAL + 1.562 CIT + 1.724 CIS-ACONITATE = organic_acids_biomass.

10.5 Protein and free amino acids

We adopt these reactions as is. In the terminology of our model, the resulting equations are:

1.15 L-ALPHA-ALANINE + 0.0959 ARG + 0.414 L-ASPARTATE + 0.0313 CYS + 1.53 GLT + 0.0445 GLY + 0.0915 HIS + 0.465 ILE + 1.51 LEU + 5.71e-05 LYS + 0.123 MET + 0.314 PHE + 0.762 PRO + 0.612 SER + 0.175 THR + 0.00409 TRP + 0.244 TYR + 0.25 VAL = protein_biomass

and

0.624 L-ALPHA-ALANINE + 0.319 ARG + 0.418 L-ASPARTATE + 0.231 CYS + 0.378 GLT + 0.740 GLY + 0.358 HIS + 0.424 ILE + 0.424 LEU + 0.380 LYS + 0.373 MET + 0.337 PHE + 0.483 PRO + 0.529 SER + 0.467 THR + 0.272 TRP + 0.307 TYR + 0.475 VAL = free_aa_biomass.

10.6 Lignin

We adopt this reaction as is. In the terminology of our model, the resulting equation is:

2.221 COUMARYL-ALCOHOL + 1.851 CONIFERYL-ALCOHOL + 1.587 SINAPYL-ALCOHOL = lignin_biomass.

10.7 Nucleic acids

We adopt this reaction as is (though note that, as discussed above, nucleotide triphosphates are not necessarily the appropriate best representation for polymerized nucleic acids). In the terminology of our model, the resulting equation is:

0.247 ATP + 0.239 GTP + 0.259 CTP + 0.258 UTP + 0.255 DATP + 0.247 DGTP + 0.268 DCTP + 0.259 TTP = nucleic_acids_biomass.

10.8 Nitrogenous compounds

We use the same nitrogenous compound weight fraction breakdown, but recalculate the stoichiometric coefficients accounting for the fact that the protein biomass, free amino acid biomass, and nucleotide biomass species each represent one gram, so that the appropriate stoichiometric coefficients of those species for the production of one total gram of nitrogenous compounds are simply the weight fractions; see Table 10.8.

Component	Species in model	unit wt (mg)	wt fraction	units/g product
Amino acids	free_aa_biomass	1000.000	0.100	0.100
Proteins	protein_biomass	1000.000	0.870	0.870
Nucleic acids	nucleic_acids_biomass	1000.000	0.030	0.030

Table 3: Nitrogenous biomass breakdown.

The resulting equation is

0.100 free_aa_biomass + 0.870 protein_biomass + 0.030 nucleic_acids_biomass = nitrogenous_biomass.

10.9 Inorganic materials

We ignore these entirely, as they play no other role in the model. (Note that even in iRS1563 the two species involved, potassium and chloride, participate only in source and sink reactions.)

10.10 Total biomass reaction

We drop the inorganic materials term (note that weight fractions now add to 0.95) and recalculate the stoichiometric coefficients, accounting for the fact that the component biomass subspecies each represent one gram; see Table 10.10.

Component	Species in model	unit wt (mg)	wt fraction	units/g product
Nitrogenous compounds	nitrogenous_biomass	1000.000	0.230	0.230
Carbohydrates	carbohydrates_biomass	1000.000	0.565	0.565
Lipids	fatty_acids_biomass	1000.000	0.025	0.025
Lignin	lignin_biomass	1000.000	0.080	0.080
Organic acids	organic_acids_biomass	1000.000	0.050	0.050

Table 4: Breakdown of total biomass.

The final equation is

> 0.230 `nitrogenous_biomass` + 0.565 `carbohydrates_biomass` + 0.025 `fatty_acids_biomass` + 0.080 `lignin_biomass` + 0.050 `organic_acids_biomass` = `total_biomass`.

Saha et al additionally incorporate an ATP cost in their overall biomass reaction, based on that used in an earlier Arabidopsis model (AraGEM; Gomes de Oliveira Dal'Molin et al. (2010)) Combining this ATP hydrolysis with a sink of total biomass, we arrive at the overall equation for biomass production and growth (`CombinedBiomassReaction`):

> 1.0 `total_biomass` + 30.0 `ATP` + 30.0 `WATER` = 30.0 `ADP` + 30.0 `Pi` + 30.0 `PROTON`

10.11 Protonation

Throughout, note that the molecular weights of species in our model may differ somewhat from those used in the iRS1563 table because of differing assumptions about protonation. The practical consequences of this difference should be limited.

10.12 Oxalate

Early drafts of the model could not produce oxalate. CornCyc indicates its production as resulting only from ascorbic acid catabolism with concomitant production of L-threonate. Recent reviews suggest this is the primary pathway of oxalate production in plant species which form calcium oxalate crystals, with the threonate ultimately being oxidized to tartrate (Franceschi and Loewus, 1995; Franceschi and Nakata, 2005; Debolt et al., 2007), though the pathways of production of soluble oxalate are less clear (Franceschi and Nakata, 2005). We found little immediate evidence that tartrate (or threonate) is formed in maize leaves at levels comparable to that of oxalate, or of pathways which could further metabolize the tartrate.

Of the three reactions in iRS1563 which could produce oxalate, only one has an associated gene: oxalate carboxylase (oxalate = formate + CO2); KEGG R00522 (EC4.1.1.2). The gene, 'ACG37538', may correspond to `GRMZM2G103512`, whose best Arabidopsis hit is `AT1G09560.1` (germin-like protein 5); it may thus be more likely to be an oxalate-consuming oxidase (Lane et al., 1993) than an oxalate carboxylase, though no function was computationally predicted for `GRMZM2G103512` in CornCyc.

We decided the available information did not allow us to accurately model oxalate production in maize. However, to retain the iRS1563 biomass equation and ensure that mass and elemental balance was preserved, we allowed production of oxalate from oxaloacetate by oxaloacetase (EC 3.7.1.1; PlantCyc `OXALOACETASE-RXN`, Plant Metabolic Network (PMN) (2014c)). This simple reaction has been observed in fungi (Hayaishi et al., 1956) but is considered unlikely to be widespread in plants (Franceschi and Nakata, 2005).

11 Plasmodesmatal transport reactions

Species allowed to be exchanged between cell types through the plasmodesmata included:

- carbon dioxide and oxygen;
- known C4 cycle metabolites alanine, aspartate, malate, PEP, and pyruvate;
- the Calvin cycle intermediates glyceraldehyde 3-phosphate and 3-phosphoglycerate;
- photorespiratory metabolites glycerate, glycolate, serine, and glycine;
- nutrients sucrose, phosphate, nitrate, ammonia, sulfate and magnesium;
- glutamate and 2-ketoglutarate;
- and cysteine and glutathione (Burgener et al., 1998).

The inclusion of compounds involved in NAD-ME C4 or C3-C4 intermediate photorespiratory carbon concentrating mechanism is not meant to suggest such a system is necessarily active in maize but merely reflects our knowledge that significant transport of those species between mesophyll and bundle sheath can occur under at least some circumstances.

References

Allen, J. F. (2003). Cyclic, pseudocyclic and noncyclic photophosphorylation: new links in the chain. *Trends in Plant Science*, 8(1):15–19.

Asada, K. (1999). The water-water cycle in chloroplasts: Scavenging of active oxygens and dissipation of excess photons. *Annual Review of Plant Physiology and Plant Molecular Biology*, 50(1):601–639.

Bartoli, C. G., Pastori, G. M., and Foyer, C. H. (2000). Ascorbate biosynthesis in mitochondria is linked to the electron transport chain between complexes III and IV. *Plant Physiology*, 123(1):335–344.

Bourgis, F., Roje, S., Nuccio, M. L., Fisher, D. B., Tarczynski, M. C., Li, C., Herschbach, C., Rennenberg, H., Pimenta, M. J., Shen, T.-L., Gage, D. A., and Hanson, A. D. (1999). S-methylmethionine plays a major role in phloem sulfur transport and is synthesized by a novel type of methyltransferase. *The Plant Cell*, 11(8):1485–1497.

Bräutigam, A. and Weber, A. P. M. (2011). Transport processes: Connecting the reactions of C4 photosynthesis. In Raghavendra, A. S. and Sage, R. F., editors, *C4 Photosynthesis and Related CO2 Concentrating Mechanisms*, number 32 in Advances in Photosynthesis and Respiration, pages 199–219. Springer Netherlands.

BRENDA (2013). Information on EC 5.1.3.2 - UDP-glucose 4-epimerase. `http://brenda-enzymes.org/enzyme.php?ecno=5.1.3.2`. October 9, 2013.

Brownleader, M., Harborne, J., and Dey, P. (1997). Carbohydrate metabolism: primary metabolism of polysaccharides. In Dey, P. and Harborne, J., editors, *Plant biochemistry*, pages 111–142. San Diego: Academic Press.

Burgener, M., Suter, M., Jones, S., and Brunold, C. (1998). Cyst(e)ine is the transport metabolite of assimilated sulfur from bundle-sheath to mesophyll cells in maize leaves. *Plant Physiology*, 116(4):1315–1322.

Debolt, S., Melino, V., and Ford, C. M. (2007). Ascorbate as a biosynthetic precursor in plants. *Annals of Botany*, 99(1):3–8.

Dörmann, P. and Benning, C. (1998). The role of UDP-glucose epimerase in carbohydrate metabolism of Arabidopsis. *The Plant Journal*, 13(5):641–652.

Douce, R. and Heldt, H.-W. (2000). Photorespiration. In Leegood, R., Sharkey, T., and von Caemmerer, S., editors, *Photosynthesis: Physiology and Metabolism*. Boston: Kluwer Academic Publishers.

Elthon, T. E. and Stewart, C. R. (1982). Proline oxidation in corn mitochondria involvement of NAD, relationship to ornithine metabolism, and sidedness on the inner membrane. *Plant Physiology*, 70(2):567–572.

Emanuelsson, O., Nielsen, H., Brunak, S., and von Heijne, G. (2000). Predicting subcellular localization of proteins based on their n-terminal amino acid sequence. *Journal of Molecular Biology*, 300(4):1005–1016.

Fettke, J., Hejazi, M., Smirnova, J., Höchel, E., Stage, M., and Steup, M. (2009). Eukaryotic starch degradation: integration of plastidial and cytosolic pathways. *Journal of Experimental Botany*, 60(10):2907–2922.

Foyer, C. and Harbinson, J. (1997). The photosythetic electron transport system: efficiency and control. In Foyer, C. and Quick, W., editors, *A Molecular Approach to Primary Metabolism in Higher Plants*. London: Taylor and Francis.

Foyer, C., Rowell, J., and Walker, D. (1983). Measurement of the ascorbate content of spinach leaf protoplasts and chloroplasts during illumination. *Planta*, 157(3):239–244.

Foyer, C. H., Bloom, A. J., Queval, G., and Noctor, G. (2009). Photorespiratory metabolism: Genes, mutants, energetics, and redox signaling. *Annual Review of Plant Biology*, 60(1):455–484.

Franceschi, V. R. and Loewus, F. A. (1995). Oxalate function and biosynthesis in plants and fungi. In Khan, S. R., editor, *Calcium oxalate in biological systems*. CRC Press.

Franceschi, V. R. and Nakata, P. A. (2005). Calcium oxalate in plants: Formation and function. *Annual Review of Plant Biology*, 56(1):41–71.

Gibson, K. J. (1993). Palmitoleate formation by soybean stearoyl-acyl carrier protein desaturase. *Biochimica Et Biophysica Acta*, 1169(3):231–235.

Gomes de Oliveira Dal'Molin C, Quek LE, Palfreyman RW, Brumbley SM, and Nielsen LK (2010). C4GEM , a genome-scale metabolic model to study C4 plant metabolism. Plant Physiology. 2010;154:1871–1885.

Hanson, A. D. and Roje, S. (2001). One-carbon metabolism in higher plants. *Annual Review of Plant Physiology and Plant Molecular Biology*, 52:119–137.

Harwood, J. L. (1996). Recent advances in the biosynthesis of plant fatty acids. *Biochimica et Biophysica Acta (BBA) - Lipids and Lipid Metabolism*, 1301(1–2):7–56.

Hayaishi, O., Shimazono, H., Katagiri, M., and Saito, Y. (1956). Enzymatic formation of oxalate and acetate from oxaloacetate. *Journal of the American Chemical Society*, 78(19):5126–5127.

Herold, A. and Lewis, D. H. (1977). Mannose and green plants: Occurrence, physiology and metabolism, and use as a tool to study the role of orthophosphate. *New Phytologist*, 79(1):1–40.

Lane, B. G., Dunwell, J. M., Ray, J. A., Schmitt, M. R., and Cuming, A. C. (1993). Germin, a protein marker of early plant development, is an oxalate oxidase. *Journal of Biological Chemistry*, 268(17):12239–12242.

Latendresse, M., Krummenacker, M., Trupp, M., and Karp, P. D. (2012). Construction and completion of flux balance models from pathway databases. *Bioinformatics*, 28(3):388–396.

Leech, R. M., Rumsby, M. G., and Thomson, W. W. (1973). Plastid differentiation, acyl lipid, and fatty acid changes in developing green maize leaves. *Plant Physiology*, 52(3):240–245.

Li-Beisson, Y., Shorrosh, B., Beisson, F., Andersson, M. X., Arondel, V., Bates, P. D., Baud, S., Bird, D., DeBono, A., Durrett, T. P., Franke, R. B., Graham, I. A., Katayama, K., Kelly, A. A., Larson, T., Markham, J. E., Miquel, M., Molina, I., Nishida, I., Rowland, O., Samuels, L., Schmid, K. M., Wada, H., Welti, R., Xu, C., Zallot, R., and Ohlrogge, J. (2013). Acyl-lipid metabolism. *The Arabidopsis Book*, page e0161.

Lopez-Calcagno, P. E., Howard, T. P., and Raines, C. A. (2014). The CP12 protein family: a thioredoxin-mediated metabolic switch? *Frontiers in Plant Science*, 5:9.

Marchler-Bauer, A. and Bryant, S. H. (2004). CD-search: protein domain annotations on the fly. *Nucleic Acids Research*, 32(suppl 2):W327–W331.

Mintz-Oron, S., Meir, S., Malitsky, S., Ruppin, E., Aharoni, A., and Shlomi, T. (2012). Reconstruction of Arabidopsis metabolic network models accounting for subcellular compartmentalization and tissue-specificity. *Proceedings of the National Academy of Sciences*, 109(1):339–344.

Munekage, Y., Hashimoto, M., Miyake, C., Tomizawa, K.-I., Endo, T., Tasaka, M., and Shikanai, T. (2004). Cyclic electron flow around photosystem I is essential for photosynthesis. *Nature*, 429(6991):579–582.

Ohlrogge, J. and Browse, J. (1995). Lipid biosynthesis. *The Plant Cell*, 7(7):957–970.

Ohshima, T., Hayashi, H., and Chino, M. (1990). Collection and chemical composition of pure phloem sap from zea mays l. *Plant and Cell Physiology*, 31(5):735–737.

Pathway Tools manual (2013). *Pathway Tools v. 17.0 user manual.* SRI International, Menlo Park, CA.

Plant Metabolic Network (PMN) (2013). CornCyc 4.0. http://pmn.plantcyc.org/CORN/organism-summary on www.plantcyc.org.

Plant Metabolic Network (PMN) (2014a). *Arabidopsis thaliana* col pathway: palmitoleate biosynthesis II. http://pmn.plantcyc.org/ARA/NEW-IMAGE?type=PATHWAY&object=PWY-5366 on www.plantcyc.org. October 16, 2014.

Plant Metabolic Network (PMN) (2014b). PlantCyc pathway: D-mannose degradation. http://pmn.plantcyc.org/PLANT/new-image?object=MANNCAT-PWY on www.plantcyc.org. October 16, 2014.

Plant Metabolic Network (PMN) (2014c). PlantCyc Reaction: 3.7.1.1. http://pmn.plantcyc.org/PLANT/NEW-IMAGE?type=REACTION-IN-PATHWAY&object=OXALOACETASE-RXN on www.plantcyc.org. October 16, 2014.

Plant Metabolic Network (PMN) (2014d). *Zea mays* mays pathway: homogalacturonan biosynthesis. http://pmn.plantcyc.org/CORN/NEW-IMAGE?type=PATHWAY&object=PWY-1061 on www.plantcyc.org. October 16, 2014.

Poolman, M. G., Kundu, S., Shaw, R., and Fell, D. A. (2013). Responses to light intensity in a genome-scale model of rice metabolism. *Plant Physiology*, 162(2):1060–1072.

Poolman, M. G., Miguet, L., Sweetlove, L. J., and Fell, D. A. (2009). A genome-scale metabolic model of Arabidopsis and some of its properties. *Plant Physiol.*, 151(3):1570–1581.

Reumann, S. and Weber, A. P. M. (2006). Plant peroxisomes respire in the light: Some gaps of the photorespiratory C2 cycle have become filled—others remain. *Biochimica et Biophysica Acta (BBA) - Molecular Cell Research*, 1763(12):1496–1510.

Rizov, I. and Doulis, A. (2000). Determination of glycerolipid composition of rice and maize tissues using solid-phase extraction. *Biochemical Society Transactions*, 28(6):586–589.

Saha, R., Suthers, P. F., and Maranas, C. D. (2011). Zea mays iRS1563: A comprehensive genome-scale metabolic reconstruction of maize metabolism. *PLoS ONE*, 6(7):e21784.

Schnarrenberger, C. (1990). Characterization and compartmentation, in green leaves, of hexokinases with different specificities for glucose, fructose, and mannose and for nucleoside triphosphates. *Planta*, 181(2):249–255.

Shanklin, J. and Cahoon, E. B. (1998). Desaturation and related modifications of fatty acids. *Annual Review of Plant Physiology and Plant Molecular Biology*, 49(1):611–641.

Shikanai, T. (2007). Cyclic electron transport around photosystem I: Genetic approaches. *Annual Review of Plant Biology*, 58(1):199–217.

Smirnoff, N. (1996). The function and metabolism of ascorbic acid in plants. *Annals of Botany*, 78(6):661–669.

Sperling, P., Schmidt, H., and Heinz, E. (1995). A cytochrome-b5-containing fusion protein similar to plant acyl lipid desaturases. *European Journal of Biochemistry*, 232(3):798–805.

Streb, S. and Zeeman, S. C. (2012). Starch metabolism in Arabidopsis. *The Arabidopsis Book*, page e0160.

Sun, Q., Zybailov, B., Majeran, W., Friso, G., Olinares, P. D. B., and van Wijk, K. J. (2009). PPDB, the plant proteomics database at Cornell. *Nucleic Acids Research*, 37(Database issue):D969–974.

Takabayashi, A., Kishine, M., Asada, K., Endo, T., and Sato, F. (2005). Differential use of two cyclic electron flows around photosystem i for driving CO2-concentration mechanism in C4 photosynthesis. *Proceedings of the National Academy of Sciences of the United States of America*, 102(46):16898–16903.

Weber, A. P. and Linka, N. (2011). Connecting the plastid: Transporters of the plastid envelope and their role in linking plastidial with cytosolic metabolism. *Annual Review of Plant Biology*, 62(1):53–77.
