# Supplementary material for: Multiscale Metabolic Modeling of C4 Plants: Connecting Nonlinear Genome-Scale Models to Leaf-Scale Metabolism in Developing Maize Leaves
Source: PLoS One. 2016 Mar 18;11(3):e0151722. doi: 10.1371/journal.pone.0151722 (PMC4807923; doi:10.1371/journal.pone.0151722)
Supplement: S2 Appendix — (PDF) [file pone.0151722.s013.pdf]

Implementation details for 'Multiscale metabolic modeling of C4 plants'

Eli Bogart Christopher R. Myers

1 Modeling interacting mesophyll and bundle sheath cells

Though in principle most small molecules can cross the plasmodesmata by diffusion [1], unrealistic concentration gradients may be required to drive high diffusive fluxes, and processes other than simple diffusion may play a role in the rapid exchanges which do occur [2]. Given this uncertainty we conservatively restricted such transport to species known or expected to be exchanged between cell types (under at least some circumstances); a complete list is given in S9 Appendix..

Net import or export of metabolites from the system was limited to the mesophyll, for gases exchanged with the intercellular airspace, or the bundle sheath, for soluble metabolites exchanged with the leaf's vascular system. Reactions were not otherwise restricted *a priori* to a particular cell type. To facilitate integration with cell-type-specific RNA data, gene associations in this model are tagged with the relevant cell type, e.g. 'bs_GRMZM2G039273' vs 'ms_GRMZM2G039273'.

2 Modeling the leaf gradient

The choice of phloem transport metabolites (other than sucrose) is a compromise. Glycine is the most abundant amino acid in maize phloem [3], and glutathione is a putative phloem sulfur transport compound [4], but many other amino acids are present in the phloem sap, and other compounds (e.g., S-methyl-methionine [4]) may play roles in phloem sulfur transport. However, we found that the available data did not adequately constrain rates of phloem transport if multiple transport species of each type were allowed, resulting in high rates of transport from the base towards the tip, against the direction of bulk flow in the phloem.

For simplicity, export of metabolites from the leaf to the rest of the plant through the phloem was neglected and net import of sucrose was not allowed. Each segment was taken to have the same total area, so that a 1 µmol m^{-2} s^{-1} rate of sucrose loading in one segment exactly balanced a 1 µmol m^{-2} s^{-1} rate of sucrose unloading in another segment.

Note that the whole-leaf model is constructed dynamically within the data-fitting code, rather than being loaded from an SBML file.

3 Integrating RNA-seq datasets

Integration of the two datasets was achieved by determining for each gene at each of point with LCM data the ratio of the average RPKM in the mesophyll (M) to the sum of the average RPKM values for mesophyll and bundle sheath ($M+B$); furthermore, we assumed that the $M/(M+B)$ ratio at the leaf base was 0.5 (based on the proteomic experiments of Majeran et al. [5], which showed only limited mesophyll-bundle sheath specificity there), and linearly interpolating to estimate $M/(M+B)$ ratios at all 15 points. For very weakly expressed genes, we did not impose cell-type specificity: where the sum of mesophyll and bundle sheath RPKM in the LCM data was less than 0.1, we assumed $M/(M+B) = 0.5$. We then divided the mean whole-leaf FPKM measurement at each point into mesophyll and bundle sheath portions according to these ratios.

To associate expression data with a reaction, data for its associated genes were summed, dividing the data for a gene associated with multiple reactions in the model equally among them. The uncertainties δ_{ij} in the objective function (eq. (3), main text) were estimated in an ad hoc way by splitting the standard deviations of the FPKM values over multiple experimental replicates according to the $M/(M + B)$ ratios and then summing the uncertainties for all genes associated with a particular reaction, imposing a minimum relative error of 0.05 and a minimum absolute uncertainty corresponding to 7.5 FPKM.

To globally rescale the expression data to be comparable to expected flux values, data for PEPC and Rubisco were compared to the enzyme activity measurements discussed below and a simple linear regression performed, yielding a scaling factor of 204 FPKM $\approx$ 1 µmol m^{-2} s^{-1} for these enzymes. All expression data were divided by this factor before solving the optimization problem. Note that this rescaling is performed for numerical convenience only. In principle, the raw data in FPKM could be supplied as input to the data-fitting algorithm; rescaling would then in effect occur separately for

each reaction, with the scaling factors determined by optimization of the s_i values in eq. 3 of the main text.

In practice, however, such an approach leads to poor performance because optimal scaling factors in that case may be large (or very small,) which can cause poor conditioning of the systems of linear equations solved in the optimization process. Applying an overall rescaling, so that the input data are, broadly speaking, more comparable in magnitude to the expected flux predictions, alleviates this issue, and the regression calculation provides a convenient heuristic for estimating an appropriate scaling factor. This process does not require, or assume, that the relationship between expression levels and fluxes (or protein levels, or enzyme activity levels) can be adequately described by a simple linear function, and the resulting scaling factor has no particular biological interpretation.

4 Incorporating enzyme activity measurements

Enzyme activities constrained by measurements in [6] were alanine aminotransferse, aspartate aminotransferase, fructose bisphosphate aldolase, glyceraldehyde 3-phosphate dehydrogenase (NADPH), glyceraldehyde 3-phosphate dehydrogenase (NADH), glutamate dehydrogenase (NADH), malate dehydrogenase (NADH), malate dehydrogenase (NADPH), PEPC, phosphofructokinase, phosphoglucomutase, phosphoglucose isomerase, phosphoglycerokinase, Rubisco, transketolase, triose phosphate isomerase, and UDP-glucose pyrophosphorylase.

For Rubisco and PEPC, enzyme data constrained the sum of the variable kinetic parameters $v_{c,\max}$ and $v_{p,\max}$ in mesophyll and bundle sheath compartments, rather than the sum of the associated fluxes (except in the calculations presented in S20 Figure, where those kinetic parameters have no meaning– there, the sum of fluxes was constrained, with the Rubisco data applied to the carboxylase, not the oxygenase, reaction.) Enzyme data in nanomole per minute per gram fresh weight was converted to micromole per second per square meter of leaf surface area assuming a fresh weight of $150\ \mathrm{g\,m^{-2}}$.

5 Calculations without kinetic laws: S20 Figure

The calculations presented in S20 Figure represent an alternative scenario where the nonlinear kinetic laws were omitted. In addition, a mathematically equivalent reformulation of the objective function was used there, which

was found to improve numerical performance in that case. The reformulation is implemented in the file `flexible_alternate.py` in the project source code.

Note also the necessary modifications to the process of incorporating the enzyme activity data in these calculations, discussed immediately above.

6 Handling reversible reactions

IPOPT requires a twice continuously differentiable objective function. We use a reformulation F' representing each absolute value $|v_{ij}|$ as the product of the flux and a parameter σ_{ij} representing its sign:

$$F'(v) = \sum_{i=0}^{N_r} \sum_{j=1}^{15} \frac{(e^{s_i}\sigma_{ij} v_{ij} - d_{ij})^2}{\delta_{ij}^2} + \alpha \sum_{i=0}^{N_r} s_i^2 \qquad (1)$$

Similarly, the enzyme activity data constraint (eq. (4), main text) was rewritten to replace absolute values in this way. Reaction rates with positive (negative) sign parameter were required to take values greater than a small negative (less than a small positive) tolerance, typically 1.0.

Choosing the σ_{ij} to optimize F' is a very large scale mixed-integer nonlinear programming problem. We arrive at an approximate solution using a heuristic method similar in spirit to that of [7], with three steps.

1. The subproblems representing each segment of the leaf are solved separately, with all scales s_i set to zero and modest upper and lower bounds on the reactions representing nutrient exchange with the phloem. Within each segment, a sign for the reversible reaction r_1 with the highest associated expression data is chosen by first setting its sign σ_1 to $+1$, finding the minimum-flux best-fitting flux distribution $\mathbf{v}^+$ ignoring the costs associated with all other reversible reactions (but including costs associated with all irreversible reactions), then finding the cost c^+ of the best-fitting flux distribution $\mathbf{v'}^+$ considering the costs of the reversible reactions with nonzero fluxes in $\mathbf{v}^+$ (temporarily setting their signs according to their values in that case.) A cost c^- is determined analogously after setting the sign σ_1 to -1 , and if $c^- < c^+$, $\sigma_1 = -1$ is chosen; otherwise, $\sigma_1 = +1$. Then the reversible reaction with the second-highest expression data r_2 is treated in the same way, considering r_1 to be irreversible.

2. When signs for all reversible reactions have been chosen at a segment, a final best-fitting flux distribution given those signs is determined.

Then the full optimization problem, combining all fifteen segments, is solved with the chosen sign parameters fixed, using those flux distributions to provide a nearly-feasible initial guess.

3. The sign-choice process in each subproblem is then solved again, fixing the scale factors s_i and rates of metabolite exchange with the phloem to those determined in the full problem. If no signs change, or if the new signs do not decrease the objective function value, fitting stops; otherwise, step 2 is repeated.

4. Finally, for each reaction i with nonzero data and maximum absolute flux less than 0.0001 at any point in the leaf model, a lower bound of $-0.99d_i$ is imposed on the term $(e^{s_i}\sigma_{ij}v_{ij} - d_{ij})$ in the objective function, for $j = 1, \ldots, 15$, and the full fifteen-segment optimization problem is solved again.

The final step addresses the observation that the optimization process occasionally converged to a solution in which a few reactions with associated data were predicted to have zero flux when a better solution with nonzero flux existed. In some cases (e.g. the $s_i = 0$ case shown in S4 Figure) this step did not lead to an overall reduction in the objective function and was omitted.

Steps 1 and 3 take between one and eight hours per segment using an AMD Opteron 6272 and may be easily parallelized across up to 15 processors. Step 2 may take up to 2 hours in the first iteration but is often faster in later iterations, when the initial guess is closer to the optimum. Typically the procedure stops after 4-5 iterations, requiring about 24 total hours of wall time using 15 processors.

7 Modified FVA procedure

Preliminary calculations showed that, for many reactions, there was a unique predicted reaction rate consistent with the optimal objective function value. However, for some of these reactions, a wide range of predicted rates was consistent with achieving an *approximately* optimal value of the objective function, while for other such reactions, relaxing the requirement of optimality to the same degree allowed only slight changes in the predicted reaction rate. To illustrate such differences, in the calculation of the upper and lower bounds for individual predicted reaction rates shown in the figures, the objective function was allowed to increase by 0.1% from its optimal value.

8 Reactions handled as special cases

The Rubisco oxygenase, Rubisco carboxylase, and mesophyll PEPC fluxes are excluded from the objective function. Instead, terms are added comparing the transcriptomic data for those enzymes to the variables which explicitly represent their activity level: for Rubisco, $v_{c,\max}$ in mesophyll and bundle sheath compartments, and for PEPC, $v_{p\max}$ in the mesophyll. Scale factors for the mesophyll and bundle sheath Rubisco activities are not constrained to be equal.

References

[1] Weiner H, Burnell JN, Woodrow IE, Heldt HW, Hatch MD. Metabolite diffusion into bundle sheath cells from C4 plants: relation to C4 photosynthesis and plasmodesmatal function. Plant Physiology. 1988 Nov;88(3):815–822.

[2] Sowiński P, Szczepanik J, Minchin PEH. On the mechanism of C4 photosynthesis intermediate exchange between Kranz mesophyll and bundle sheath cells in grasses. Journal of Experimental Botany. 2008 Apr;59(6):1137–1147.

[3] Ohshima T, Hayashi H, Chino M. Collection and chemical composition of pure phloem sap from *Zea mays* L. Plant and Cell Physiology. 1990 Jan;31(5):735–737.

[4] Bourgis F, Roje S, Nuccio ML, Fisher DB, Tarczynski MC, Li C, et al. S-methylmethionine plays a major role in phloem sulfur transport and is synthesized by a novel type of methyltransferase. The Plant Cell. 1999 Aug;11(8):1485–1497.

[5] Majeran W, Friso G, Ponnala L, Connolly B, Huang M, Reidel E, et al. Structural and metabolic transitions of C4 leaf development and differentiation defined by microscopy and quantitative proteomics in maize. The Plant Cell. 2010 Nov;22(11):3509–3542.

[6] Wang L, Czedik-Eysenberg A, Mertz RA, Si Y, Tohge T, Nunes-Nesi A, et al. Comparative analyses of C4 and C3 photosynthesis in developing leaves of maize and rice. Nature Biotechnology. 2014 Oct;32:1158–1165.

[7] Lee D, Smallbone K, Dunn WB, Murabito E, Winder CL, Kell DB, et al. Improving metabolic flux predictions using absolute gene expression data. BMC Systems Biology. 2012 Jun;6(1):73.
