# Supplementary material for: Multiscale Metabolic Modeling of C4 Plants: Connecting Nonlinear Genome-Scale Models to Leaf-Scale Metabolism in Developing Maize Leaves
Source: PLoS One. 2016 Mar 18;11(3):e0151722. doi: 10.1371/journal.pone.0151722 (PMC4807923; doi:10.1371/journal.pone.0151722)
Supplement: S1 Table — In addition to the reactions listed, transport capacities of pyruvate, PEP, alanine, aspartate and malate across the plasmodesmata and pyruvate, PEP, malate and oxaloacetate across the chloroplast inner membrane could limit this rate; the model currently associates no genes with these transport reactions. (PDF) [file pone.0151722.s015.pdf]

reaction	name in model	associated genes
malate dehydrogenase (NADP)	`MALATE_DEHYDROGENASE_NADP__RXN_chloroplast`	1
alanine aminotransferase	`ALANINE_AMINOTRANSFERASE_RXN`	10
aspartate aminotransferase	`ASPAMINOTRANS_RXN`	7
NAD-malic enzyme	`EC_1_1_1_39`	2
NADP-malic enzyme (cytosol)	`MALIC_NADP_RXN`	4
NADP-malic enzyme (chloroplast)	`MALIC_NADP_RXN_chloroplast`	2
PEPCK	`PEPCARBOXYKIN_RXN`	6
PPDK	`PYRUVATEORTHOPHOSPHATE_DIKINASE_RXN_chloroplast`	2
adenylate kinase	`ADENYL_KIN_RXN_chloroplast`	6
pyrophosphatase	`INORGPYROPHOSPHAT_RXN_chloroplast`	2

S10 Table. Detailed parameters contributing to the effective PEP regeneration rate. Reactions in the genome-scale model which contribute to the effective maximum PEP regeneration capacity, and numbers of genes associated with each. In addition to the reactions shown here, transport capacities of pyruvate, PEP, alanine, aspartate and malate across the plasmodesmata and pyruvate, PEP, malate and oxaloacetate across the chloroplast inner membrane could limit this rate; the model currently associates no genes with these transport reactions.
